# Perspectives for the reconstruction of 3D chromatin conformation using single cell Hi-C data

**DOI:** 10.1371/journal.pcbi.1009546

**Published:** 2021-11-18

**Authors:** Pavel I. Kos, Aleksandra A. Galitsyna, Sergey V. Ulianov, Mikhail S. Gelfand, Sergey V. Razin, Alexander V. Chertovich

**Affiliations:** 1 Friedrich Miescher Institute for Biomedical Research, Basel, Switzerland; 2 Faculty of Physics, Lomonosov Moscow State University, Moscow, Russia; 3 Semenov Federal Research Center for Chemical Physics, Moscow, Russia; 4 Skolkovo Institute of Science and Technology, Moscow, Russia; 5 Institute of Gene Biology, Russian Academy of Sciences, Moscow, Russia; 6 Institute for Information Transmission Problems (Kharkevich Institute), Moscow, Russia; 7 Faculty of Biology, Lomonosov Moscow State University, Moscow, Russia; University of Maryland School of Pharmacy, UNITED STATES

## Abstract

Construction of chromosomes 3D models based on single cell Hi-C data constitute an important challenge. We present a reconstruction approach, DPDchrom, that incorporates basic knowledge whether the reconstructed conformation should be coil-like or globular and spring relaxation at contact sites. In contrast to previously published protocols, DPDchrom can naturally form globular conformation due to the presence of explicit solvent. Benchmarking of this and several other methods on artificial polymer models reveals similar reconstruction accuracy at high contact density and DPDchrom advantage at low contact density. To compare 3D structures insensitively to spatial orientation and scale, we propose the Modified Jaccard Index. We analyzed two sources of the contact dropout: contact radius change and random contact sampling. We found that the reconstruction accuracy exponentially depends on the number of contacts per genomic bin allowing to estimate the reconstruction accuracy in advance. We applied DPDchrom to model chromosome configurations based on single-cell Hi-C data of mouse oocytes and found that these configurations differ significantly from a random one, that is consistent with other studies.

## Author summary

Reconstruction of 3D configuration of chromosomes based on single cell Hi-C data (contact matrix) constitutes an important way to understand the specific features of genome packaging in individual cells. For this purpose, we develop method DPDchrom based on dissipative particle dynamics. Two sources of the contact dropout, contact radius change and random contact sampling, have different impact to the reconstruction accuracy suggesting unequal value of contacts. We found the exponential dependence of the reconstruction accuracy on the number of contacts per genomic bin. We reconstructed entire nucleus of mouse using single cell Hi-C data and found significant dissimilarity of conformations reconstructed using experimental and shuffled data. These findings open perspectives to study 3D arrangement of cis-regulatory elements orchestrating transcription program.

## Introduction

The levels of DNA packaging, such as chromatin compartments [1], topologically associating domains (TADs) [2], and loops, are largely conserved between different cell types [3]. However, the conformation of chromosomes in individual cells varies significantly [4].

There are two main experimental approaches to study the spatial chromatin organization: methods based on chromosome conformation capture (for a review see [5]) and microscopy techniques (fluorescent *in situ* hybridization [6], live-cell imaging [7], *etc*.). For comprehensive description of various methods in 3D genomics see recent review [8].

The *all vs. all* version of chromosome conformation capture, Hi-C, remains a key source of knowledge about the chromatin structure averaged over the population of cells [1, 9]. The common steps of the experimental procedure include chromatin cross-linking, enzymatic fragmentation of DNA, proximity ligation, and massive parallel sequencing [1]. Recently developed single-cell and single-nucleus Hi-C approaches capture contacts in individual cells or nuclei [4, 10–12], opening up a unique opportunity to bridge the gap between Hi-C and microscopy.

Numerous theoretical models have been proposed to describe the spatial organization of chromatin [13]. We split all theoretical models into two general groups of mechanistic and reconstructive approaches.

A mechanistic approach is based on a hypothesis on what orchestrates the chromosome structure, which is typically supported by experimental findings. The aim is to reproduce structural features of chromatin, such as loops, TADs, and compartments. For example, the widely studied loop extrusion model reproduces peaks at TAD corners and stripes at TAD boundaries [14]. Another model introduces specific interactions between beads that results in TADs and compartments [15, 16].

A reconstructive approach uses Hi-C contact maps as an input. The chromatin structure can be represented as either a single conformation [17] or multiple conformations [18, 19]. In the former case, a 3D conformation is constructed that minimizes the difference between the experimental contact matrix and the matrix reconstituted from that conformation. In the latter case, an obtained set of conformations should collectively match the input Hi-C contact map. At that, there is no evidence that any particular conformation obtained by these methods occurs *in vivo*.

Reconstructive models for population-based Hi-C search for multiple solutions and cannot reject wrong conformations [20]. Nevertheless, statistical findings obtained by these methods have helped to understand the population-averaged principles of chromatin folding [21].

**Fig 1:**
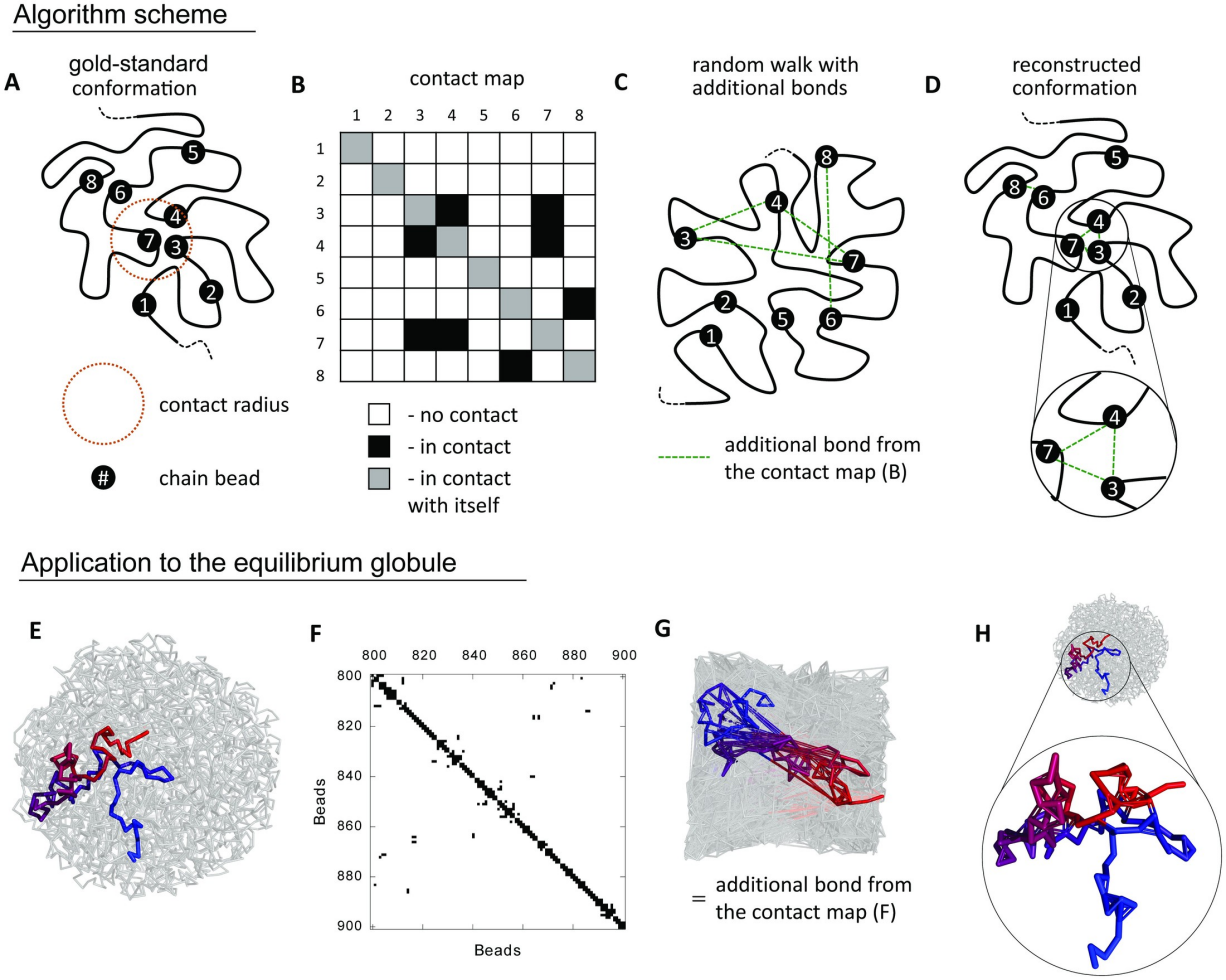
The algorithm scheme (A-D) and a demonstration of its application to the equilibrium globule (E-H). **A**. The worm-like view of a chain fragment. The numbers denote beads selected for the demonstration. **B**. The contact map corresponds to the gold-standard conformation (A). **C**. The chain in a random walk conformation with additional bonds from the contact map (B). **D**. The chain after equilibration (C), *a.i*. the reconstructed conformation. **E**. The equilibrium globule with *l*_0_ = 0.5, semitransparent gray. The chain segment 800–900 is highlighted and colored from red to blue. **F**. The contact map for the highlighted segment from (E), the contact radius is set to *r*_*contact*_ = 0.8. **G**. The initial conformation of the chain generated as a random walk in a cubic cell. Additional bonds from the contact map are added to the system, most of them are overstretched before equilibration. The same chain region as in (E) is highlighted. **H**. The equilibrated system of (G). The magnifying pan shows the highlighted region from (E) with additional bonds.

Several techniques for the reconstruction of chromatin conformations from single-cell Hi-C data have been proposed based on polymer models [4, 12] and the Bayesian inference [22]. The Bayesian inference methods are derived from statistical properties of scHi-C and do not use physical assumptions about the structures, and thus are beyond the scope of this study [23]. The bead-and-spring models underlie many modeling methods to describe the behavior of coarse-grain polymers, Fig 1. These methods include the method proposed by Stevens *et al*. [12] (hereinafter the Stevens method, it was utilized in the Dip-C/hickit software for single-cell modelling [24]), method based on classical molecular dynamics (CMD) [25, 26], and the updated version of DPDchrom [27] (see Data Availability) relying on dissipative particle dynamics (DPD) [28].

What is the actual reconstruction accuracy and how the conformation type and contact reduction affect the reconstruction accuracy remain uncertain. To address these questions, we

1. compared the results obtained by different reconstruction methods;
2. estimated the reconstruction accuracy on gold-standard polymer models;
3. studied two sources of contacts dropout: contact radius variation and random contact sampling;
4. applied DPDchrom to experimental single cell Hi-C contact maps.

## Results

### DPDchrom is a versatile tool for the reconstruction of single-cell Hi-C conformations

We developed a pipeline DPDchrom based on the DPD scheme [28], to reconstruct 3D conformations of chromosomes. DPDchrom initializes the chromosomes as a set of bead-and-spring polymer chains surrounded by solvent beads. Then an initial random conformation is equilibrated to minimize the energy of overstretched bonds and turns into a conformation corresponding to the provided single cell Hi-C contact map. DPDchrom and the Stevens method use interaction potentials softer than those in CMD, hence preventing vitrification during equilibration. The significant limitation of the Stevens method is its ability to generate only coil-like conformations with additional constraints, whereas DPDchrom can also form a globular conformation due to the presence of explicit solvent.

In DPDchrom, we rely on the general idea that the reconstruction of a 3D conformation from a given set of contacts is equivalent to the rearrangement of a random conformation by iterative bringing together contacting polymer beads. We have determined the set of parameters that are optimal for the reconstruction of a chromatin conformation.

The constraints, such as connectivity along the chain and additional bonds from the list of spatial neighbors, define a family of multiple conformations. Families may have different sizes. A family size is defined by conformational freedom of the units (mostly, free of additional bonds) and soft nature of the entire system (bends, twists, etc.). It is impossible to fully describe (enumerate) all the conformations from the family, because the conformational space grows very rapidly with an increase in the degrees of freedom. Its size can be qualitatively assessed through the reconstruction accuracy. A high reconstruction accuracy means a small variation between possible conformations and, therefore, represents a small volume of the conformational space suitable for a given contact map.

### Benchmarking on *in silico* conformations

Single-cell Hi-C produces a readout of a single conformation of chromosomes in an individual nucleus. Each contact represents two genomic regions that are spatially close to each other. Experimentally it means formation of a detectable ligation product. We reproduced the Hi-C experiment *in silico* and benchmarked the possible reconstruction accuracy comparing with gold-standard conformations.

The gold-standard conformation is an array of bead coordinates of a single linear bead-spring polymer chain, with the length *N* = 4096 beads. Unless otherwise stated, we have performed calculations on a polymer globule, a polymer chain collapsed in a single droplet (minimizing surface with a solvent). We have also used the Moore curve—analytical space-filling curve densely sweeping the 3D space and polymer solutions.

A globule corresponds to a chromosome conformation at a resolution lower than 10 kb. A bead from the model represents not only the chromatin fiber but also the solvent and proteins. The diameter of a bead from the model equals the distance between ends of a 10 kb chromatin segment. Therefore, the effective chromatin concentration is close to 100%, corresponding to globular conformation. Thus, at the resolution 10 kb or lower, it is recommended to consider a chromosome or the entire nucleus as a polymer globule.

Firstly, we generate a gold-standard polymer conformation (globule, the Moore curve or polymer solution). Next, we determine the pairs of beads that are in a spatial contact by applying a contact radius threshold *r*_*contact*_. Then, we use this set of contacts as an input for the reconstruction algorithm. We create a random walk conformation with additional bonds, which we take from the contact map. Due to the possibility of self-intersections (see Materials and methods), conformation is rearranged, and additional overstretched bonds take on their normal length. Finally, we compared the reconstructed 3D conformation with the gold-standard 3D conformation by calculating the reconstruction accuracy (see the Modified Jaccard Index in Materials and methods). This design is schematically outlined in Fig 1 and the details of each step can be found in Materials and methods.

### Comparison of methods

We computed the reconstruction accuracy of each method applied to *in silico* gold-standard conformations. The detailed descriptions of the methods and their implementation are given in S1 Appendix.

**Fig 2:**
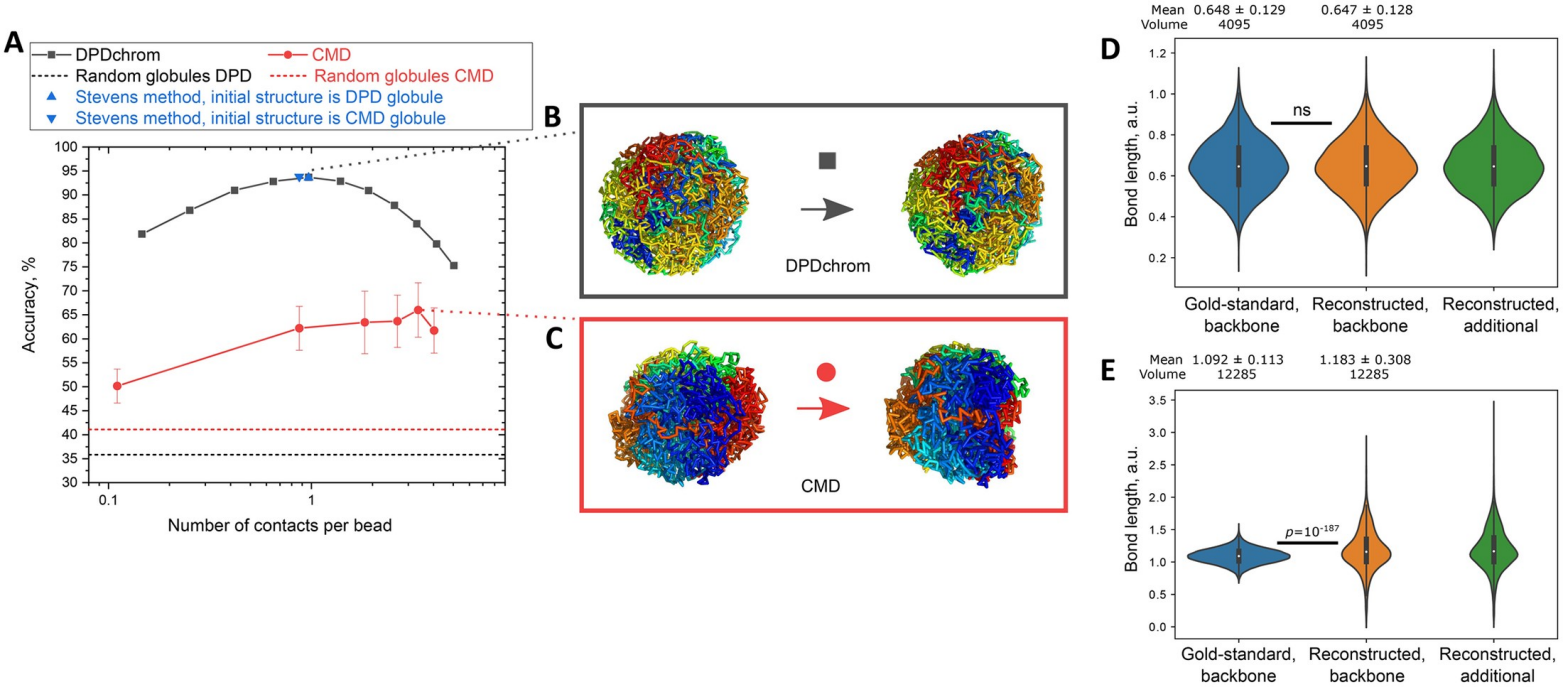
**A**. Dependence of the reconstruction accuracy on the number of contacts per bead for DPDchrom, CMD, and the Stevens method. The dashed lines represent the degree of similarity of two random polymer globules obtained by DPD and CMD, respectively. **B, C**. The gold-standard (left) and the best reconstructed (right) conformations. The chains are colored by rainbow from the first bead to the last one. **D, E**. The bond length distributions of the gold-standard conformation and those reconstructed by DPDchrom (D) and CMD (E). For the reconstructed conformations, the backbone bonds and the additionally introduced bonds from the gold-standard contacts are represented separately.

To operate with more biologically intuitive parameters, we converted the contact radius *r*_*contact*_ into the averaged number of detected contacts per bead (S6 Fig). We define the number of contacts per conformation unit (genome bin or chain bead) as the total number of unique contacts divided by the total number of these units in the conformation. This number is an intuitive representative of the quality of a single cell Hi-C dataset (Fig 2A). That is, the larger is the bin size of single cell Hi-C data, more contacts per bin are detected.

Both DPD and CMD can be used in the generative mode to create artificial structures (further utilized as gold-standard ones). To create a realistic testing scheme, we reconstructed each algorithm on the random conformations generated by itself. However, the Stevens method is not generative, thus we applied it to CMD- and DPD-produced stuctures with adjusted spatial scales. We normalized the coordinates to the radius of gyration of the entire system and then calculated the respective reconstruction accuracy.

For the CMD and DPDchrom, we show the gold-standard and the reconstructed systems with the highest reconstruction accuracy in Fig 2B and 2C. The reconstruction accuracy for DPDchrom is significantly higher than that for CMD at all tested specific numbers of contacts per bead (Fig 2A). This difference is likely to be due to the soft interaction potentials of DPDchrom. At high number of contacts, the Stevens method demonstrates the reconstruction accuracy similar to that of DPDchrom (blue triangles in Fig 2A). We attribute this observation to the fact that the interaction potential of the Stevens method is similar to the potential of DPDchrom, S1 Appendix.

To compare the properties of the gold-standard and reconstructed conformations, we calculated the distributions of bond lengths for the backbone and additional bonds separately (Fig 2D and 2E). The expectation for a well equilibrated and perfectly reconstructed system is that the distribution of bond lengths before and after reconstruction should be almost the same. This holds for DPD, but not for CMD (Fig 2D and 2E). The two-sided Wilcoxon signed-rank test demonstrates that the median length of DPDchrom-reconstructed conformations does not differ from that for the gold-standard conformation (both equal 0.65, *p*-value = 0.69, non-significant, Fig 2E). The same test shows that the median bond lengths for CMD gold-standard and reconstructed conformations differ significantly with the *p*-value of 10^−187^ (the medians are 1.09 and 1.16, respectively, Fig 2D). We further confirmed these findings by the Kolmogorov-Smirnov test (S1 Table and S1 Appendix). We propose that this difference in the distributions indicates a kinetic trap and represents poor reconstruction and system equilibration by CMD (see Discussion). We hence conclude that DPD outperforms CMD for the conformation reconstruction.

### The reconstruction accuracy depends on the polymer volume fraction

**Fig 3:**
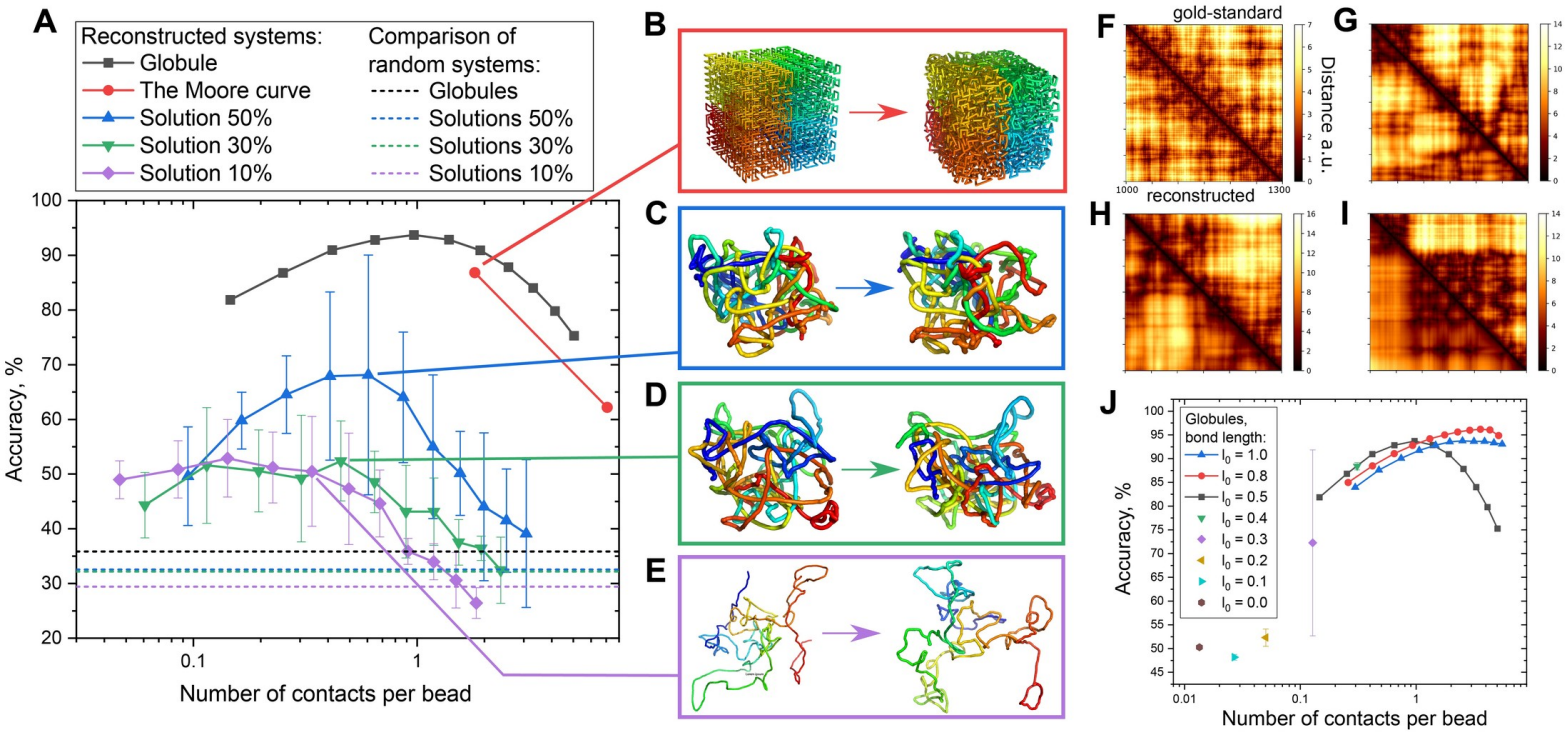
**A**. Dependency of the reconstruction accuracy on the number of contacts per bead for studied polymer systems: globule, the Moore curve, and solutions (50, 30, and 10% of the polymer volume fraction). **B-E**. Visualized results of reconstruction: left—the gold-standard conformation, right—the reconstructed conformation. The systems corresponding to the visualized conformations are marked by lines in (A). Solvent particles are hidden. **F-I**. Comparison of the gold-standard and the reconstructed distance maps. A short segment [1000, 1300] is shown for better visual perception. The top right triangle corresponds to the gold-standard conformation and the left bottom triangle corresponds to the reconstructed conformation. F—fractal globule (B), G—50% polymer concentration (C), H—30% polymer concentration D, and I—10% polymer concentration (E). **J**. Dependence of the reconstruction accuracy on the number of contacts per bead.

We note that the true conformation of the genome in the nucleus is unknown, and both genome length and nucleus size can vary for different species. Thus, to study the limits of reconstruction applicability, we varied the polymer volume concentration. We compared the globule (≈ 100%), the Moore curve [29] (≈ 100%), and solutions with the polymer volume concentrations of 50%, 30%, and 10% (Fig 3A). For the globule, we tested the impact of the contact radius on reconstruction accuracy.

For dense structures, the globule and the Moore curve, the ensemble averaging was not required because variation of the contact radius yielded a smooth response (Fig 2A). Along with decrease in the polymer volume concentration, the number of contacts decreases, and the system becomes more sparse and dynamic. To account for significant fluctuations, we averaged each point for solutions over five independent runs (Fig 3A). Fig 3B–3E features visualizations of the reconstruction results. For visual convenience, some conformations (Fig 3C–3E) were smoothed in coordinates within windows of 20 beads along the chain. For each represented structure, we plot the distance maps for a segment of 300 beads starting from bead number 1000, Fig 3F–3I.

To determine the optimal set of parameters and the limits of applicability of DPDchrom, we created gold-standard conformations using the initial bond length *l*_0_ from the set [0.0, 0.1, ‥0.5, 0.8, 1.0], (Fig 3J). We computed the contact matrix using the contact radius *r*_*contact*_. For three values of the initial bond length *l*_0_ = 0.5, 0.8, 1.0, we varied the contact radius *r*_*contact*_ within [0.5, 1.0] in increments of 0.05. For the remaining values of *l*_0_, we used one value *r*_*contact*_ = *l*_0_, because for *r*_*contact*_ = 0.5, 0.8, 1.0, the maximum accuracy is achieved at *r*_*contact*_ = *l*_0_. At *l*_0_ ≤ 0.3 the system lose chain phantomness, because successive beads are too close to each other that prevents chain intersection. In this case, the reconstructed conformation cannot reach the gold-standard conformation and is kinetically trapped.

In Fig 3J, considering three initial bond lengths *l*_0_ = 0.5, 0.8, and 1.0, we see that the maximum accuracy is reached at *l*_0_ = 0.8 (the red curve). Notably, when the number of contacts per bead is 0.3, the reconstruction with the initial bond length *l*_0_ = 0.5 yields higher accuracy than at *l*_0_ = 0.8, 1.0, probably due to smaller radius resulting in the most valuable contacts.

### The reconstruction accuracy exponentially depends on the number of contacts per bead

In a single cell Hi-C experiment, the contact map is sparse and usually has the resolution lower than 10 Kb due to the contacts dropout and the limitations caused by the distribution of restriction fragments in the genome [26, 27]. We next studied the influence of contact sampling based on the gold-standard globule conformation.

**Fig 4:**
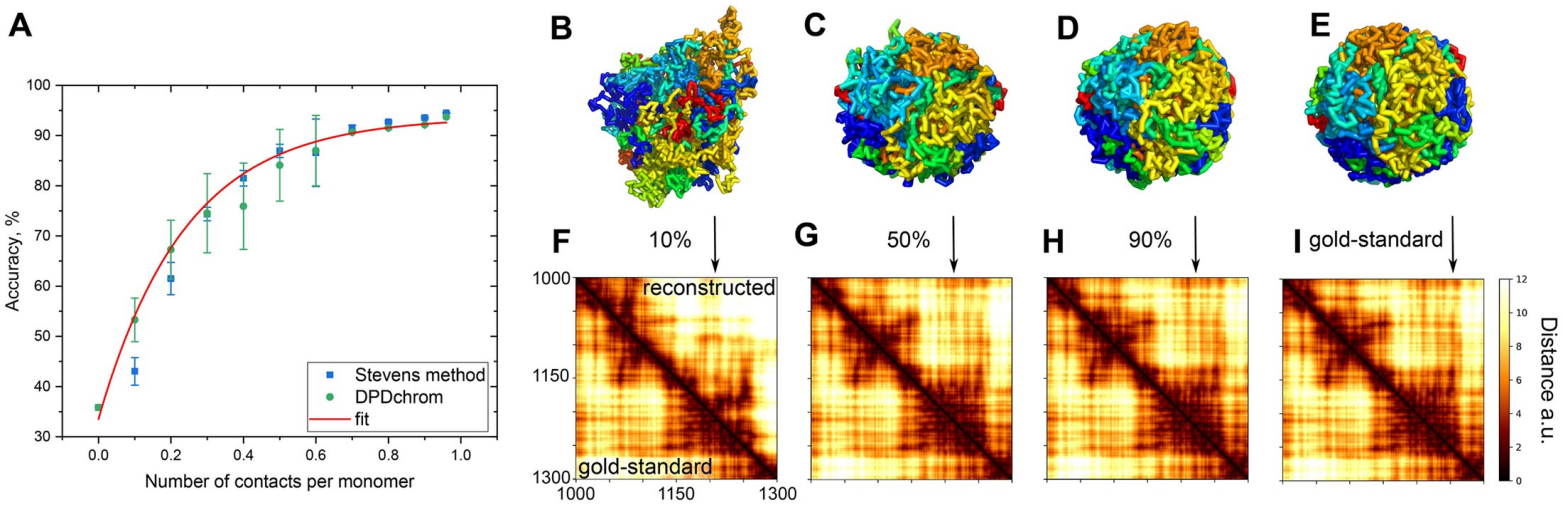
**A**. Dependence of the reconstruction accuracy on the number of contacts per bead. DPDchrom and the Stevens method were applied to reconstruct globule using a fraction of contacts within the range [0, 100]%. The red line represents the exponential fit for DPDchrom. **B-D**. Reconstructed 3D conformations using 10%, 50%, 90% of contacts, colored by rainbow from the first to the last bead. **E**. Gold-standard 3D conformation, colored as in (B-D). **F-H**. Distance maps of the gold standard (lower left triangle) and the reconstructed (upper right triangle) conformations using 10%, 50%, 90% of contacts. **I**. Distance map of the gold-standard conformation (both triangles).

To simulate the contacts dropout, we sampled contacts randomly, keeping 0−100% contacts and performed ten independent runs for each fraction and for each reconstruction method (Fig 4A). Both methods demonstrate almost similar reconstruction accuracy.

The system can fluctuate, and the beads on the surface of the structure are more mobile than the beads inside it. This leads to natural limitations on the achievable reconstruction accuracy. For the globule, where the number of contacts per bead reaches maximum, we obtained the maximal reconstruction accuracy *A*_*max*_ ≈ 93.7% using DPDchrom and the Stevens method. Decrease of contact number results in exponential decrease in reconstruction accuracy, which could be approximated with the universal (i.e. method-independent) expression (Eq 1).

$$\begin{array}{c} Accuracy=\left(1-A*e^{-x/\beta }\right)*A_{max} \end{array}$$ (1)

where *x* is the number of contacts per bead, *A* is the coefficient representing available similarity of two structures minus the similarity of two random structures (1 − *A* = 0.358, see Fig 4A), *β* is the numerical coefficient related to physical properties of conformation and reconstruction methodology, and *A*_*max*_ ≈ 93.7% is the maximal achievable reconstruction accuracy. For DPDchrom we found *A* = 0.642 and *β* = 0.239.

The pattern of exponential decrease in the reconstruction accuracy is also seen when comparing distance maps for systems with 90, 50, and 10% of retained contacts. At 10%, the reconstructed distance map qualitatively reproduces some motives of the gold-standard map. At 50% and 90%, it is almost impossible to visually find differences with the gold-standard map, Fig 4B–4I.

### DPDchrom application to experimental data

In order to demonstrate DPDchrom applicability to experimental data, we downloaded eleven single-nucleus Hi-C datasets [10, 11] (S2 Table and S1 Appendix) and performed *de novo* analysis with the One-Read Based Interactions Annotation (ORBITA) approach (see Data Availability), which produces contact maps with minimal contribution of experimental artefacts that can lead to self-inconsistent conformations. We compared the number of contacts for each individual cell reported in the papers with those in ORBITA analysis and found good correspondence, S1 Fig.

We extracted the number of contacts per bin (10 Kb) from the datasets for the entire genome and each chromosome separately. We note that for real single-cell datasets, the choice of resolution is arbitrary and covers a wide range from 10 Kb to 1 Mb [10, 11]. Importantly, the selection of resolution determines the number of contacts per bead, and hence, limits the achievable accuracy of reconstruction. The dependence of the number of contacts per bead on the resolution is power-law because of the power-law dependence of the contact probability on the genome distance [1]. We focused on resolutions of 20, 100, and 200 Kb to test the robustness of DPDchrom.

**Fig 5:**
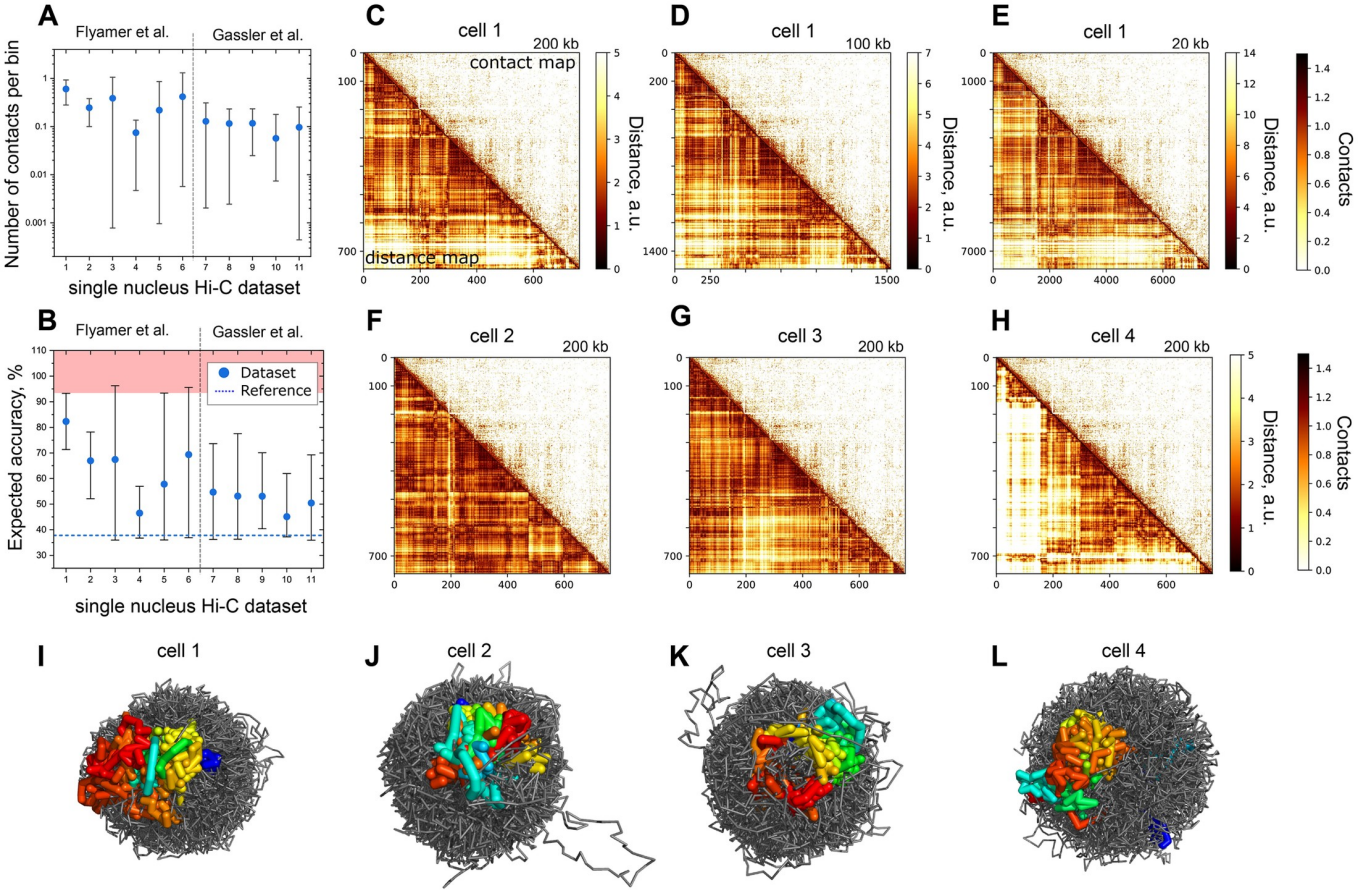
**A**. Distributions of the number of contacts per bin for single cells from 11 datasets. **B**. Distributions of the expected accuracy are calculated for the datasets (A) using Eq 1. **C-E**. The lower left triangle is the distance map of the reconstructed chromosome 4 from cell 1. The upper right triangle is the experimental contact map of the same chromosome. The resolutions are 200 Kb, 100 Kb, and 20 Kb. **F-H**. Comparison of the distance maps of chromosome 4 from cells 2, 3, 4 with the merged contact map at resolution 200 Kb. **I-L**. Chromatin conformations of cells 1–4 at resolution 200 Kb. Chromosome 4 is colored rainbow from start to end. The remaining chromatin fiber is gray.

The number of contacts per genome bin varies in a wide range, from 0.001 to 1 for different experimental runs (Fig 5A). We applied the exponential dependence (Eq 1) to these numbers of contacts per bin to estimate the expected accuracy for eleven downloaded single-nucleus datasets (Fig 5B). Interestingly, there are few cells with more than 0.975 contacts per bin (the red zone in Fig 5B), and their expected accuracy is 93.7% according to our prediction.

Finally, we focused on the conformation reconstruction for one particular cell type, mouse oocytes with non-surrounded nucleolus (#3, S2 Fig) [10], because it contains the cell with the largest number of contacts. These cells contained four copies of each chromosome, the maternal and paternal diploid genomes. However, the snHi-C protocol does not recognize chromosome copies, thus we consider the system with each chromosome type represented as a single polymer chain.

To probe different setups, we reconstructed cell 1 at the resolutions of 200 Kb, 100 Kb, and 20 Kb, Fig 5C–5E. We compared the distance maps of the reconstructed structures with the merged contact map of all 44 cells of the same cell type. The distance maps at different resolutions are qualitatively similar to each other, demonstrating that we reproduced the general structure at all considered scales and DPDchrom correctly handled cases of a large number of contacts.

We compared the contact map of chromosome 4 with the merged contact map at resolution 200 Kb, and the merged contact map with the shuffled one for control. The contact map of the reconstructed conformation is more similar to the merged map than shuffled ones, (S7A–S7D Fig). For resolutions of 100 Kb and 20 Kb, we performed only the visual inspection, because of different resolutions of these matrices and representation of four copies of chromosomes as single chains. However it reveals general similarity as well.

To test the robustness of metrics IMJ to assess similarity of two maps, we calculated the Spearman correlation coefficient for the same pairs of maps: merged experimental contact map vs single reconstructed contact map and merged experimental contact map vs shuffled reconstructed contact map. Essentially we got similar results when using IMJ (S7F–S7G Fig).

Next, we considered four randomly selected cells and reconstructed the conformation of chromatin structure for these entire cells at the resolution of 200 Kb, Fig 5C and 5F–5H. We compared the contact maps with each other and with shuffled ones and observed high similarity of the reconstructed contact maps, except that of cell 4. The contact map of cell 4 is more similar to the shuffled contact maps than to the contact maps of other cells. But the shuffled maps are more similar to each other than to the contact map of cell 4. These findings support the hypothesis of non-randomness of the chromatin conformation, despite strong cell-to-cell variability (S7E Fig) [27].

To visualize the DPDchrom results, we plotted the 3D conformations of chromatin of entire cells (Fig 5I–5L). Most of the chromosomes are well segregated, but in cell 4 parts of colored chromosome spread within the entire nucleus, suggesting that its conformation differs from that of the others.

We tried to reconstruct 3D conformation using bulk data. For this purpose we used well-studied system of *α*-globin region in mouse embryonic stem cells (mESCs) and differentiated erytroid cells (ECs). It was shown, that *α*-globin region takes hairpin conformation in ECs, whereas in the mESCs there is no such a structure [30, 31]. We took two capture-C contact maps of that particular region and sampled 10% contacts with the weights corresponding to the number of contacts. We also assumed that every genome bin can have two contacts at most with other bins. We couldn’t compare reconstructed structure with the sampled contact map. Instead we compared distance maps of reconstructed structures with each other (S8A Fig). We calculated significance of the difference between three groups: mESCs-mESCs, ECs-ECs, and mESCs-ECs. We found that between mESCs-ECs and mESCs-mESCs, the two sample KS test shows *p*-value = 10^−3^. Other pairs show *p*-value <0.05 by the two sample KS test (S8 Fig). However, by an eye inspection we see that loop appear in ECs more often (S8C Fig).

Great candidate to test applicability of DPDchrom is oligopaint experiment. In this experiment, consecutive segments of a single chromosome tagged with fluorescent probes. It opens an opportunity to have a distance map of that chromatin region and directly compare it with the distance map of reconstructed conformation. To test the applicability of DPDchrom, we used data from two recent works [32, 33].

**Fig 6:**
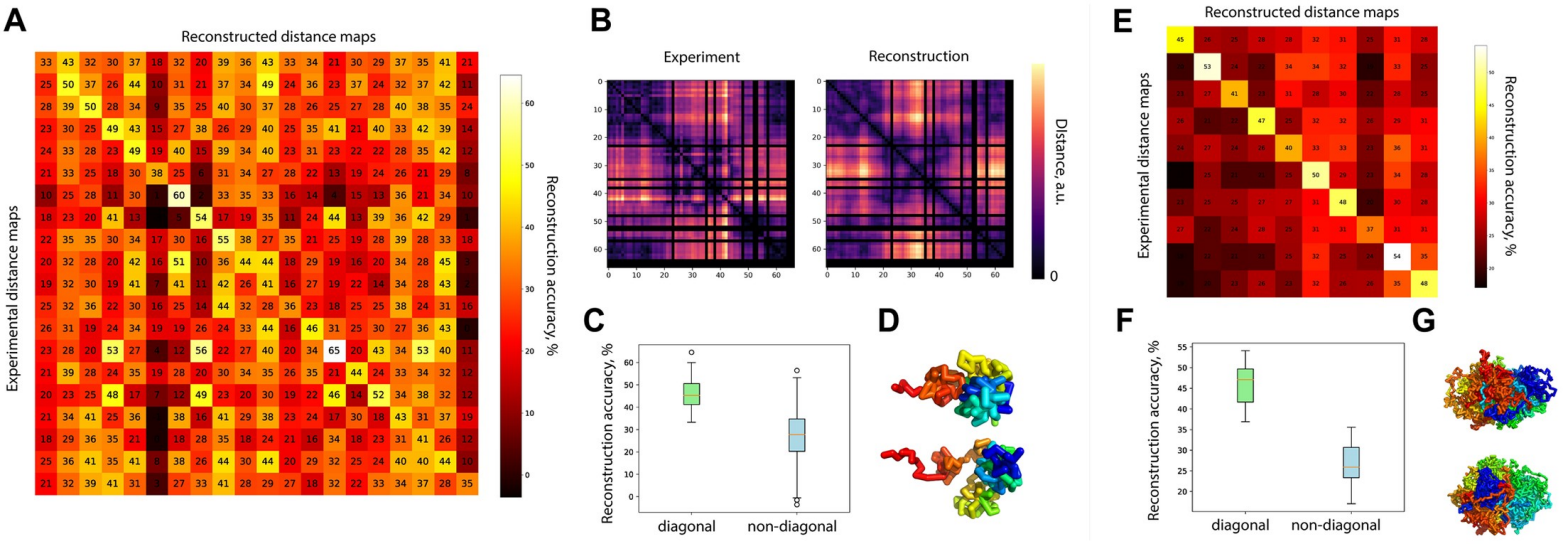
**A**. Pairwise comparison of distance maps corresponding to experimental data [32] and reconstructed 3D model. In a cell unit, a number indicates reconstruction accuracy, %. **B**. Example of experimental and reconstructed distance maps corresponding to 28−30Mb of chromosome 21. Distance map of the reconstructed structure masked with the same pattern as the experimental one has. **C**. Boxplots of reconstruction accuracies, diagonal elements correspond to matching structure and map, such as i-th experimental dataset and i-th reconstructed structure. Non-diagonal elements correspond to i-th experimental dataset, j-th reconstructed structure. **D**. Examples of two reconstructed 3D structures from (A). **E**. Pairwise comparison similar to (A) but for another experimental data [33]. **F**. Boxplots of reconstruction accuracies, similar to (C). **G**. Examples of two reconstructed 3D structures from (E).

From the first work [32], we randomly selected 20 distance maps and performed reconstruction of 3D conformations. We compared all-vs-all and represented result as a matrix (Fig 6A). Elements on the diagonal correspond to a similarity rate between experimental distance map and distance map of the structure reconstructed from the contact map, created from that experimental distance map. Non-diagonal elements correspond to similarity between 3D structure and some other experimental data set. In the Fig 6B, there is an example of experimental and reconstructed distance maps, horizontal and vertical black lines correspond to the missing experimental data and we created the mask of missing data and applied it to the reconstructed map for the proper comparison. In the Fig 6C, reconstructed structure has higher similarity to it’s experimental map (diagonal) rather than to any other map (non-diagonal). Two-sample KS test shows *p*-value = 10^−10^. In the Fig 6D, we represented two examples of reconstructed conformations. Since we consider the same region, despite cell-to-cell variability, some motifs are repeated between different conformations. Also the chain length is rather short (200 beads), that limits the variety of possible conformations and results in a higher similarity between structures.

From another work [33] sharing oligopaint data, we randomly selected 10 distance maps corresponding to chromosome 2. We created contact matrix and then reconstructed 3D conformation using DPDchrom. We compared experimental distance matrices with the ones of reconstructed conformations (Fig 6E). In this particular case, the chain is longer than in the Fig 6A–6D, that leads to a more pronounced difference between diagonal and non-diagonal similarity rates (Fig 6F). The two sample KS test shows *p*-value = 10^−13^. We also represented two examples of 3D conformations. From this research [33] we performed reconstruction for the segment of chromosome 21 (S8D–S8F Fig). We obtained similar results such as in the Fig 6. The reconstructed accuracy takes values in the range 40−60%, cell-to-cell variability we estimate as ≈30%, similar to what we see comparing random conformations of a chain.

## Discussion

In this work, we determined the dependence of reconstruction accuracy on the quality of the single cell Hi-C data. No such methods were proposed, and we fill this gap by the comprehensive study of the accuracy on the quality of simulated single-cell Hi-C data. The dependence can be found in Eq 1 and further used for quantitative estimation of the reconstruction accuracy on real data. We note that this expression is valid not only for DPDchrom, but also for the Stevens method when the number of contacts per bead more than 0.3. When the expected accuracy exceeds the upper limit of 93.7%, we propose it a good practice to increase the resolution of scHi-C (e.g. if the estimation was done for 10 Kb, one can try 5 Kb instead). If a finer structure is not required, the reconstruction accuracy cannot exceed 93.7% according to our results.

We studied two major sources of contact dropout in the system, reducing the contact radius and the contact dropout. An important observation from our simulations, when the number of contacts per bead is 0.15, reducing the contact radius leads to the accuracy of 80% (Fig 3A), whereas contact dropout leads to the accuracy of 60% (Fig 4A). We speculate that reducing of the contact radius keeps the closest contacts which are the structure-defining contacts, whereas contact sampling keeps contacts randomly.

Using an updated method DPDchrom, we have studied conformations of chromosomes from mouse oocytes. Although four copies of chromosomes were represented as a single chain, its conformation could be distinguished from a conformation with random contacts having the same scaling. We speculate that the maternal and paternal sets of chromosomes usually have similar structures, but this needs further research [10, 24, 34].

We have also studied the reconstruction possibilities using experimental bulk capture-C data and single cell oligopaint data. Reconstruction using bulk capture-c of *α*-globin chromatin segment shows mild differences between mESCs and ECs. For the oligopaint data we see a prominent difference even between conformations of the same genomic region in different cells. DPDchrom demonstrates accuracy of 35% for capture-C data and of 50% for oligopaint data (Fig 6).

Benchmarking on artificial data shows that DPDchrom significantly outperforms CMD in reconstruction tasks. It also outperforms the Stevens method in the globule reconstruction at low numbers of contacts per bead (Fig 4A).

New methods of chromatin conformation capture constantly emerge, including novel single-cell techniques, such as scSPRITE [35]. Methods that distinguish contacts of sister chromatids for a population of cells, such as SisterC [34] and scsHi-C [36], are of particular interest. We believe that with the development of single-cell Hi-C versions of these methods, DPDchrom would be effectively used to reconstruct conformations of diploid nuclei and genomes containing several copies of chromosomes.

To summarize, DPDchrom is a powerful method for single-cell chromatin structure reconstruction, with comprehensively described and supported by in silico benchmarking usage on the real data scHi-C data. We find it important that DPDchrom is able to reproduce the entanglements and the reptation dynamics of a polymer chain [37–39]. Thus, DPDchrom can be extended to describe dynamic properties of the chromatin fiber, that allows to study transcriptional regulation.

## Materials and methods

### Performing reconstruction

Using DPD and CMD, we can create conformations, not just reconstruct them, whereas the Stevens method has been developed only to reconstruct conformations. Excluding the Moore curve, which is analytical space-filling curve and perfect fractal [29], we used DPD and CMD to create the gold-standard conformations. We put the polymer chain in appropriate environment conditions: poor solvent for the globule and good solvent for the solutions [40], details are given in S1 Appendix. Hence, we have constructed the following gold-standard conformations: globule by CMD, globule and solutions by DPD, and the Moore curve.

To compare the reconstruction accuracies for the polymer solutions, the Moore curve, and the globule, we set the length of the polymer chain to 4096 beads. This length was selected because the Moore curve grows as 8^x^ (where *x* is an integer number) to fill the space densely. The length of 8^4^ = 4096 beads is a good compromise between 8^3^ = 512, which is too short to study the reconstruction accuracy, and 8^5^ = 32768 which is computationally prohibitive.

To equilibrate the bond lengths, the system needs to self-intersect, because it has a complex connectivity pattern and because knots could have been formed during construction of the initial random walk conformation. The initial bond length (*l*_0_) and its stiffness (*k*) are selected to allow the polymer chain to self-intersect [41]. In case of CMD, a steep repulsion term of the volume interaction potential still prevents self-intersection when multiple bonds intend to cross a single bond (Eq. 1 in S1 Appendix). Such an area becomes overcrowded and the beads stuck. Further rearrangement of the system is kinetically unavailable, and the system cannot reach the state with a lower potential energy, this effect called vitrification [42]. Increasing the bond length decreases the energy barrier of intersection but limits the reconstruction accuracy.

The distributions of bond lengths and distances between non-bonded beads yield *l*_0_ = 1.1 in CMD (S2 and S3 Figs) and *l*_0_ = 0.5 in DPDchrom (S4 and S5 Figs). In single cell Hi-C, the contact radius is unknown. According to the algorithm, we calculated a set of contact matrices for each gold-standard conformation using different contact radii (*r*_*contact*_). For DPD, the range was [0.5, 1.0] with the step of 0.05, and for CMD the range was [1.0, 1.5] with the step of 0.1.

We studied the dependence of the reconstruction accuracy on the contact radius. We converted the contact radius into the number of contacts per bead (S6 Fig). The optimal contact radius equals the equilibrium bond length (the initial bond length *l*_0_ is extended due to the repulsion volume potential). Increase or decrease of the contact radius leads to the degradation of the reconstruction accuracy. A mismatch between the contact radius and the equilibrium bond length makes some beads closer in the reconstructed conformation than in the gold-standard one. For sparse solutions, the best accuracy is observed when the contact radius is smaller than the equilibrium bond length, Fig 3A.

### Gold-standard conformations and sets of contacts

We considered the following gold-standard conformations: equilibrium globule, the Moore curve, polymer chain in a neutral solvent in confinement with polymer volume concentrations of 10%, 20%, and 50%. We deduced contacts between chain beads from individual structures. For each gold-standard conformation, we fixed the coordinates of all beads and the connectivity of the system, Fig 1A and 1E. Then, we calculated the contact map. For that, we defined the threshold value of the contact radius *r*_*contact*_. For any pair of beads *i* and *j*, a contact occurs if *r*_*ij*_ ≤ *r*_*contact*_, Fig 1A. Thus, we constructed a contact map corresponding to a given gold-standard 3D conformation, Fig 1B and 1F.

### Input random conformation

As the next step, we generated a random walk in a cubic box that served as the input for the reconstruction of the gold-standard conformation. Similar to chromatin volume concentration in the nucleus [43], we fixed the polymer concentration as the ratio between the number of beads in chains and the total number of beads in the system.

The differences between the gold-standard and the initial conformations are the beads’ positions and the connectivity. In the initial conformation, we introduce additional bonds corresponding to contacts in the gold-standard conformation. Many of these additional bonds are overstretched, Fig 1C and 1G.

#### Reconstruction of conformations

We reconstructed the system using DPDchrom, CMD, and the Stevens method. The initial conformation of the random walk is rearranging because the overstretched bonds tended to regain their normal length (as backbone bonds in gold-standard conformations). The polymer chain in simulations is phantom, two strands of the chain can pass through each other when the energy gain is larger than the barrier induced by the excluded volume. Thus, chain segments can come in close proximity as in the gold-standard conformation even if the connectivity topologically prohibits such rearrangement, Fig 1D and 1H.

### The choice of simulation parameters

#### CMD

We used the canonical NVT-ensemble [44] with implicit solvent taken into account through the Lennard-Jones potential. Parameters for the Lennard-Jones potential are the depth of the potential well *ϵ*^*CMD*^ = 1.0, the bead size *σ*^*CMD*^ = 1.0, and the cutting radius of interaction $r_{c}^{CMD}=2.5$. This set of parameters is a usual choice for poor solvent conditions yielding globular state of a polymer chain. The default Nose-Hoover thermostat was used [45].

The spring stiffness constant was fixed at *k*^*CMD*^ = 40. At $l_{0}^{CMD}=1.0$, the system stucks in a non-equilibrium state because the chain cannot cross itself. At $l_{0}^{CMD}=1.5$, the chain can cross itself but beads have much more entropy than at $l_{0}^{CMD}=1.0$. Free bond length $l_{0}^{CMD}$ was varied in the range [1.0, 1.5] with step of 0.1. Which was supported also by the distributions of spatial distances between the non-bonded beads of the polymer chain (S2 and S3 Figs).

#### DPD

All parameters are given in arbitrary units. In Eq. 3–5 in S1 Appendix, the cutting radius *r*_*c*_ equals 1.0, in Eq. 6 in S1 Appendix the bond stiffness *k* equals 40, similar to CMD. An unperturbed bond length $l_{0}^{DPD}$ was varied in the range [0.0, 1.0] (S4 and S5 Figs).

The repulsion parameters are *a*_*ij*_ = 55 and *a*_*ii*_ = 25, so the Flory-Huggins parameter *χ* equals 8.58. This specifies poor solvent conditions, where the equilibrium polymer state is a globule (see SI). The repulsion parameter *a*_*ii*_ = 25 provides self-intersections of the polymer chain yielding short equilibration time by removing knots and entanglements.

Due to the soft potential, Eq. 3 in S1 Appendix, the integral time step equals 0.04, that is about 1 magnitude larger than in CMD.

### The Modified Jaccard Index

To compare two 3D polymer conformations, we compared their distance maps. A distance map is a symmetric matrix *N* × *N* containing Euclidean distances between all polymer beads. Position of the center of mass and rotation of the conformation have no impact on the distance map. The distance matrix is also invariant to mirror reflection of the conformation and our method does not distinguish such conformations. For visual representation, we used reconstructed and gold-standard conformations with the same chirality, performing mirror reflection when necessary.

We introduced *IMJ* (the modified Jaccard index) [46] to measure similarity between distance maps. Let *D* and *D*′ be two distance matrices corresponding to the pair of structures being compared. We calculate its symmetric and asymmetric parts: *A*_−_ = (*D* − *D*′)/2 and *A*_+_ = (*D* + *D*′)/2. *IMJ* is the ratio ||*A*_−_||/||*A*_+_||, where ||Matrix|| is a simple euclidean norm ($\left||Matrix|\right|=\sqrt{a_{11}^{2}+a_{12}^{2}+‥+a_{21}^{2}+\ldots }$, where *a*_*ij*_ is a matrix element).

To measure the reconstruction accuracy, we normalized *IMJ* as *A* = (*IMJ*_*r*_ − *IMJ*_12_)/*IMJ*_*r*_ * 100%, where *IMJ*_*r*_ = 0.378 corresponds to *IMJ* for two random symmetric matrices (zero level) and *IMJ*_12_ is *IMJ* for systems 1 and 2. For the two equal symmetric matrices (identical structures) *IMJ*_12_ = 0 and the accuracy is *A* = 100%, whereas for the two random symmetric matrices, *IMJ*_12_ = 0.378 and the accuracy is *A* = 0%. We created matrices 20000 × 20000 and repeated it 10 times. This value is the same for smaller matrices, but the results have large standard deviations. In order to compare two structures obtained by methods with different spatial scales (DPDchrom, CMD, and Stevens method), the distance matrix was divided by the radius of gyration of the polymer chain.

## Supporting information

S1 Fig Number of contacts per individual nucleus. **A**. Flyamer et al. 2017 datasets. **B**. Gassler et al. 2017 datasets.
(PDF)

S2 Fig Distance distributions. Distributions of distances between bonded and non-bonded beads of the chain. CMD, *l*_0_ = 2.0.
(PDF)

S3 Fig Distance distributions. Distributions of distances between bonded and non-bonded beads of the chain. CMD, *l*_0_ = 1.1.
(PDF)

S4 Fig Distance distributions. Distributions of distances between bonded and non-bonded beads of the chain. DPD, *l*_0_ = 0.5.
(PDF)

S5 Fig Distance distributions. Distributions of distances between bonded and non-bonded beads of the chain. DPD, *l*_0_ = 1.0.
(PDF)

S6 Fig Corresponding between specific number of contacts per bead and cutting radius. Dependence of specific number of contacts per polymer bead on cutting radius for the systems with various polymer concentration and for globule with various initial bond length.
(PDF)

S7 Fig Comparison of contact maps. Accuracies were calculated according to the definition from The Modified Jaccard Index. **A-D**. “Proper” means the similarity of the contact map of reconstructed chromosome 4 from the cells 1 − 4 and merged experimental contact map of all datasets. “Shuffled” means the similarity of the shuffled contact map of reconstructed chromosome 4 from the cells 1 − 4, keeping the initial amount of contacts on the sub-diagonal, and merged experimental contact map. **E**. “Proper” corresponds to the similarity of contact maps of reconstructed conformations to each other. “Shuffled” corresponds to the similarity of contact maps of the reconstructed conformations to shuffled ones. Shuffled were prepared in the same way as in a-d. Group of the outliers correspond to the cell 4. Its contact maps is more similar to the shuffled one than to other cells. **F-I**. The same as (A-D) but instead of IMJ we used the Spearman correlation coefficient.
(PDF)

S8 Fig Reconstruction using capture-C data. **A**. Pairwise comparison of distance maps corresponding to experimental capture-C data [30, 31] and reconstructed 3D model. In a cell unit, a number indicates reconstruction accuracy, %. **B**. Summary of matrix from (A). Boxplots of reconstruction accuracies. *p*-values correspond to the two sample KS test. **C**. Examples of reconstructed 3D conformations of ECs and mESCs. **D**. Pairwise comparison of distance maps corresponding to experimental oligopaint data [33] corresponding to chromosome 21. **E**. Boxplots of reconstruction accuracies for (D), similar to (B). **F**. Examples of two reconstructed 3D structures from (D).
(PDF)

S1 Table Statisical tests. Kolmogorov-Smirnov tests for DPD and CMD distributions from Fig 2D and 2E.
(PDF)

S2 Table List of single nucleus Hi-C datasets. (PDF)

S1 Appendix Details of simulation methods and analysis. Description of DPD, CMD, Stevens method, insights to polymer solutions, information on statistical tests, description of single-nucleus Hi-C data processing, and ORBITA protocol.
(PDF)
