# Supplementary material for: Perspectives for the reconstruction of 3D chromatin conformation using single cell Hi-C data
Source: PLoS Comput Biol. 2021 Nov 18;17(11):e1009546. doi: 10.1371/journal.pcbi.1009546 (PMC8601426; doi:10.1371/journal.pcbi.1009546)
Supplement: S1 Fig — A. Flyamer et al. 2017 datasets. B. Gassler et al. 2017 datasets. (PDF) [file pcbi.1009546.s001.pdf]

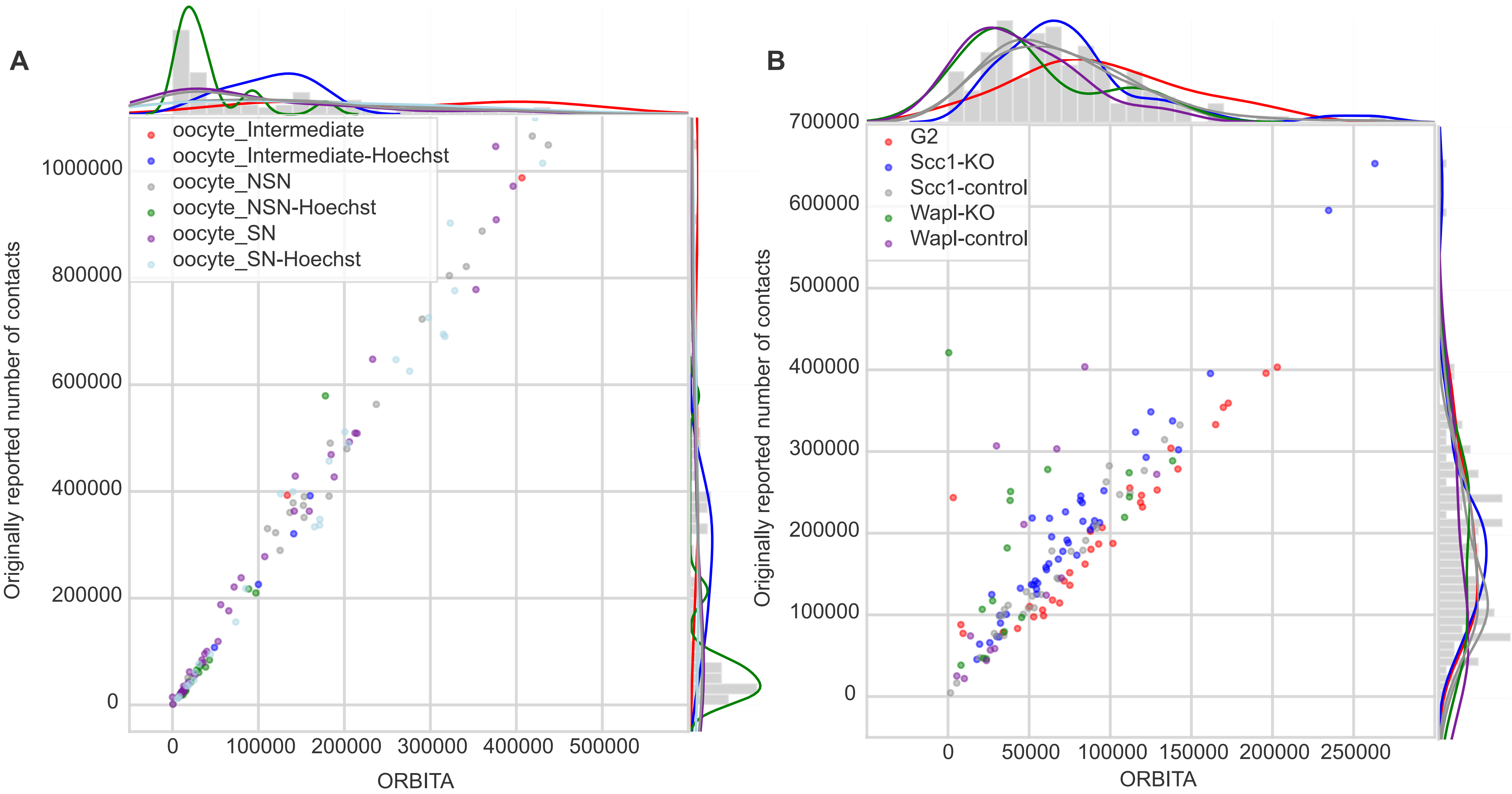
A
oocyte_Intermediate
oocyte_Intermediate-Hoechst
oocyte_NSN
oocyte_NSN-Hoechst
oocyte_SN
oocyte_SN-Hoechst
Originally reported number of contacts
0
200000
400000
600000
800000
1000000
0
100000
200000
300000
400000
500000
ORBITA
B
G2
Scc1-KO
Scc1-control
Wapl-KO
Wapl-control
Originally reported number of contacts
0
100000
200000
300000
400000
500000
600000
700000
0
50000
100000
150000
200000
250000
ORBITA
