# Supplementary figures and images for: Perspectives for the reconstruction of 3D chromatin conformation using single cell Hi-C data
Source: PLoS Comput Biol. 2021 Nov 18;17(11):e1009546. doi: 10.1371/journal.pcbi.1009546 (PMC8601426; doi:10.1371/journal.pcbi.1009546)

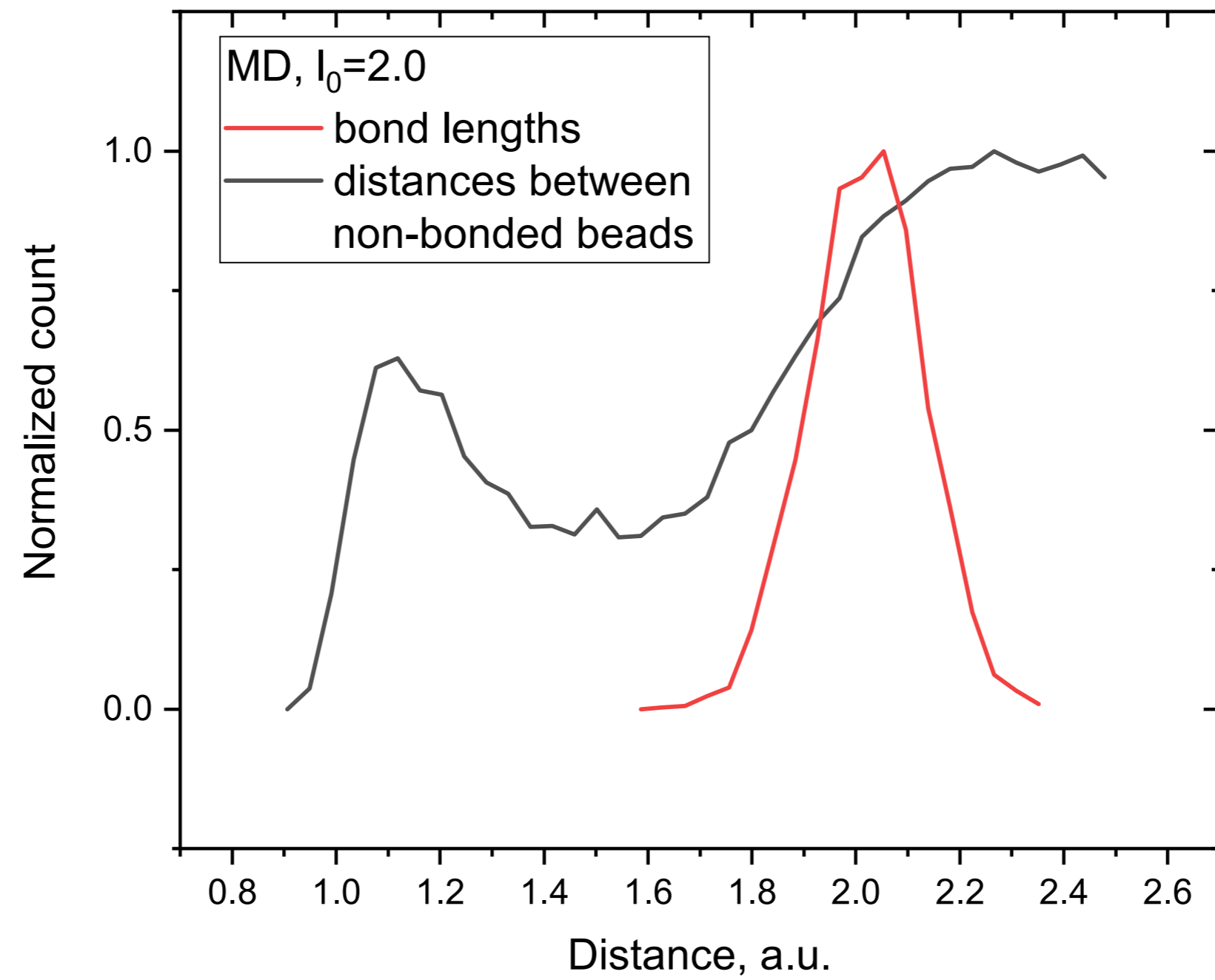

Supplement: S2 Fig — Distributions of distances between bonded and non-bonded beads of the chain. CMD, l0 = 2.0. (PDF) [file pcbi.1009546.s002.pdf]

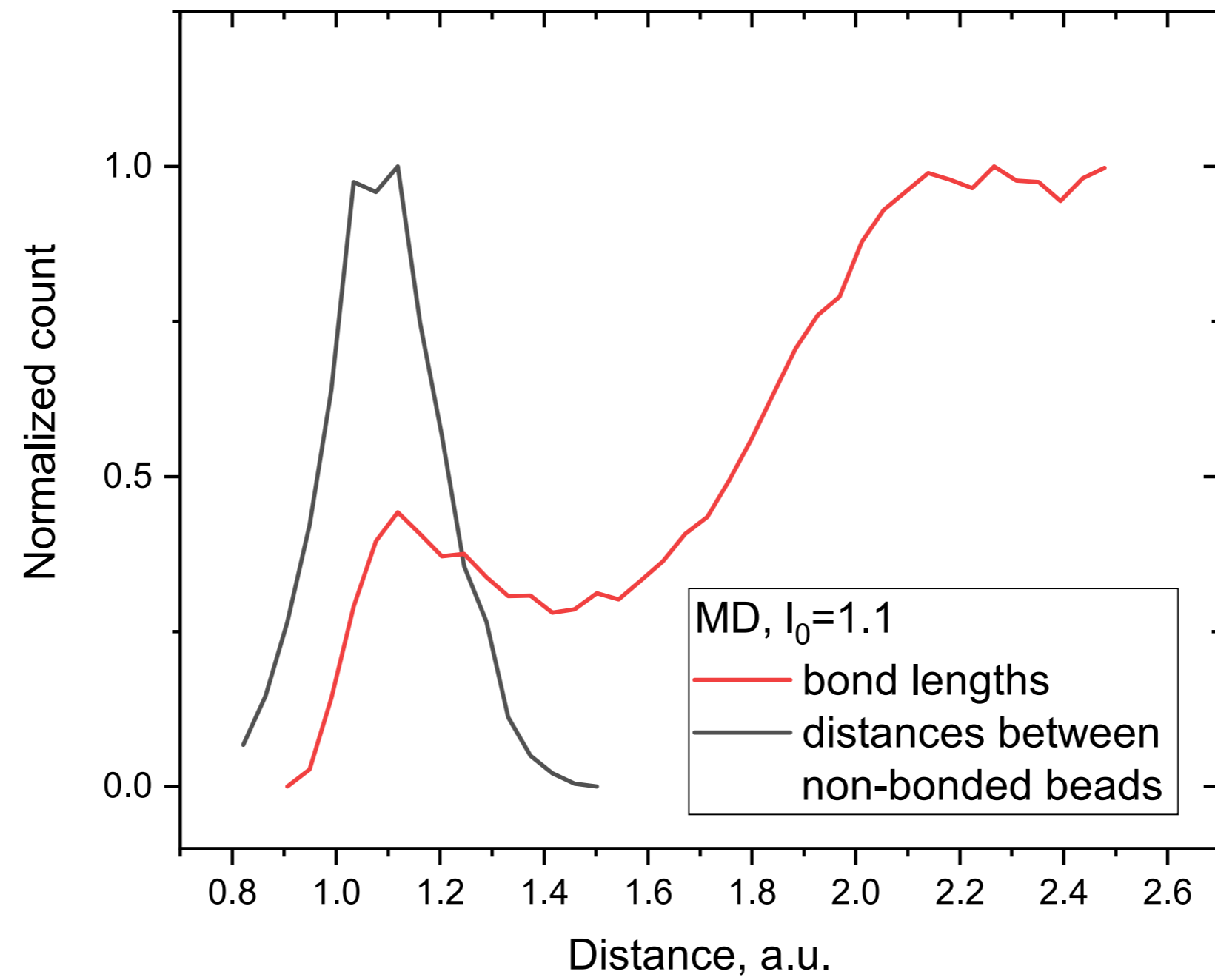

Supplement: S3 Fig — Distributions of distances between bonded and non-bonded beads of the chain. CMD, l0 = 1.1. (PDF) [file pcbi.1009546.s003.pdf]

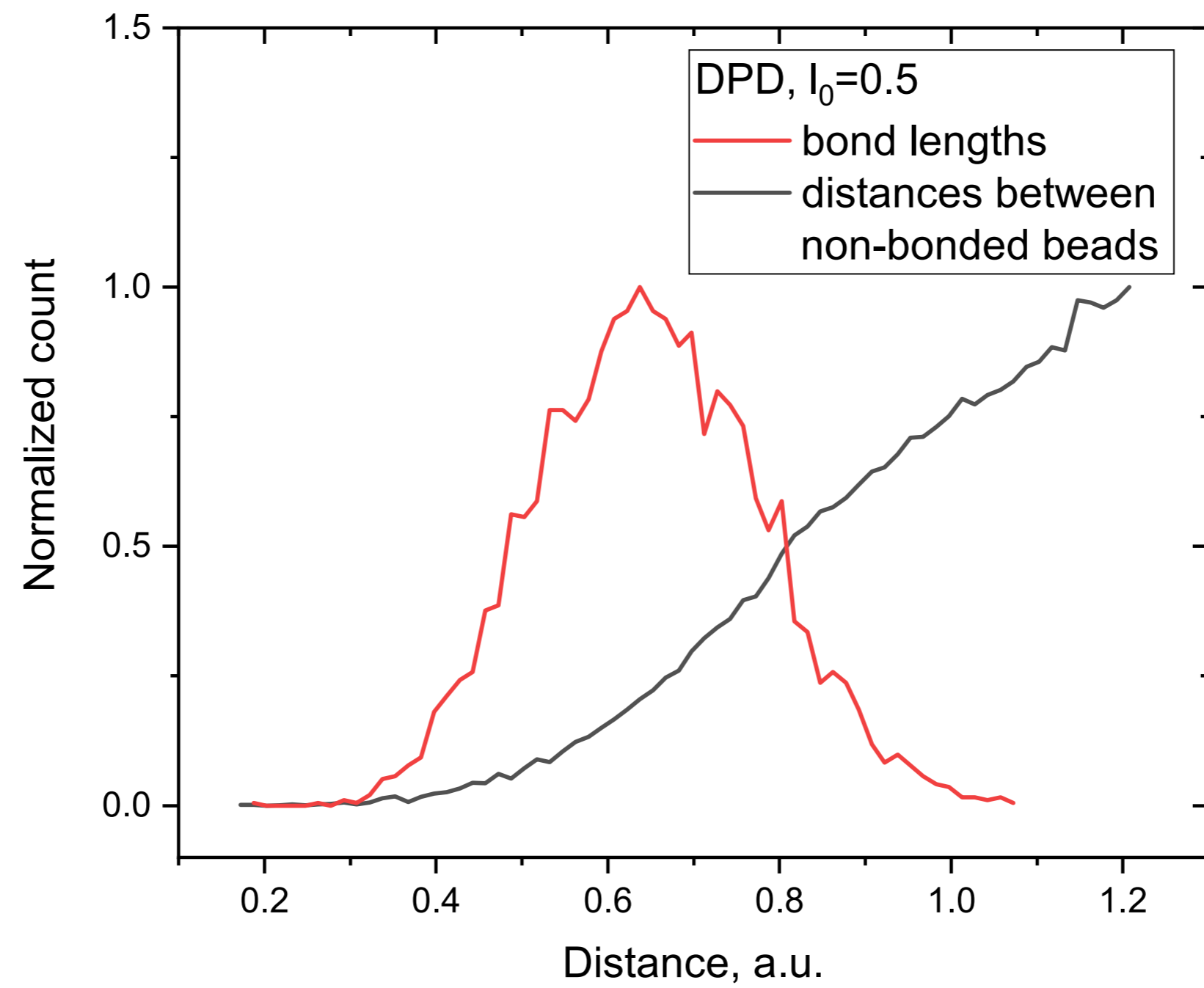

Supplement: S4 Fig — Distributions of distances between bonded and non-bonded beads of the chain. DPD, l0 = 0.5. (PDF) [file pcbi.1009546.s004.pdf]

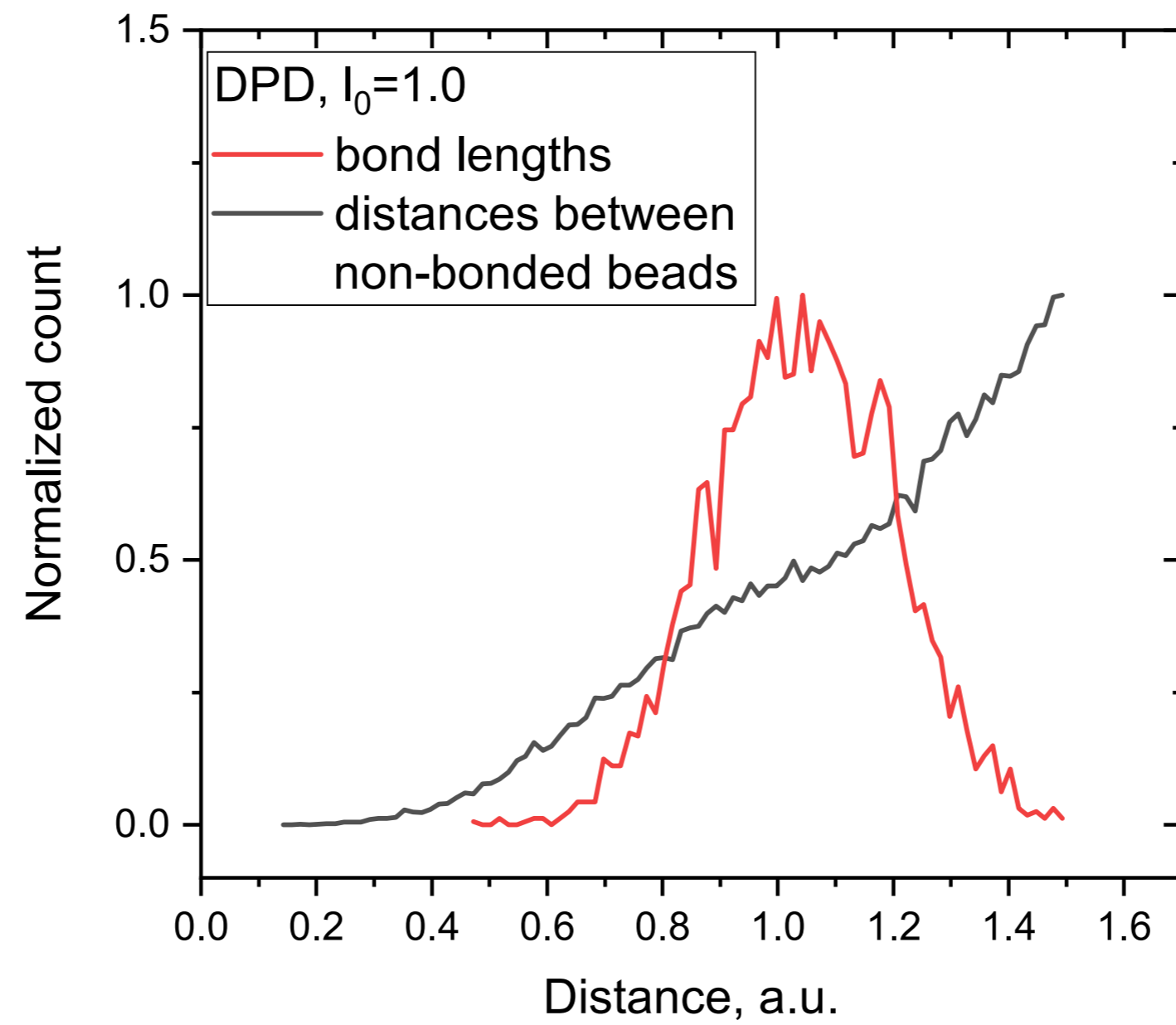

Supplement: S5 Fig — Distributions of distances between bonded and non-bonded beads of the chain. DPD, l0 = 1.0. (PDF) [file pcbi.1009546.s005.pdf]

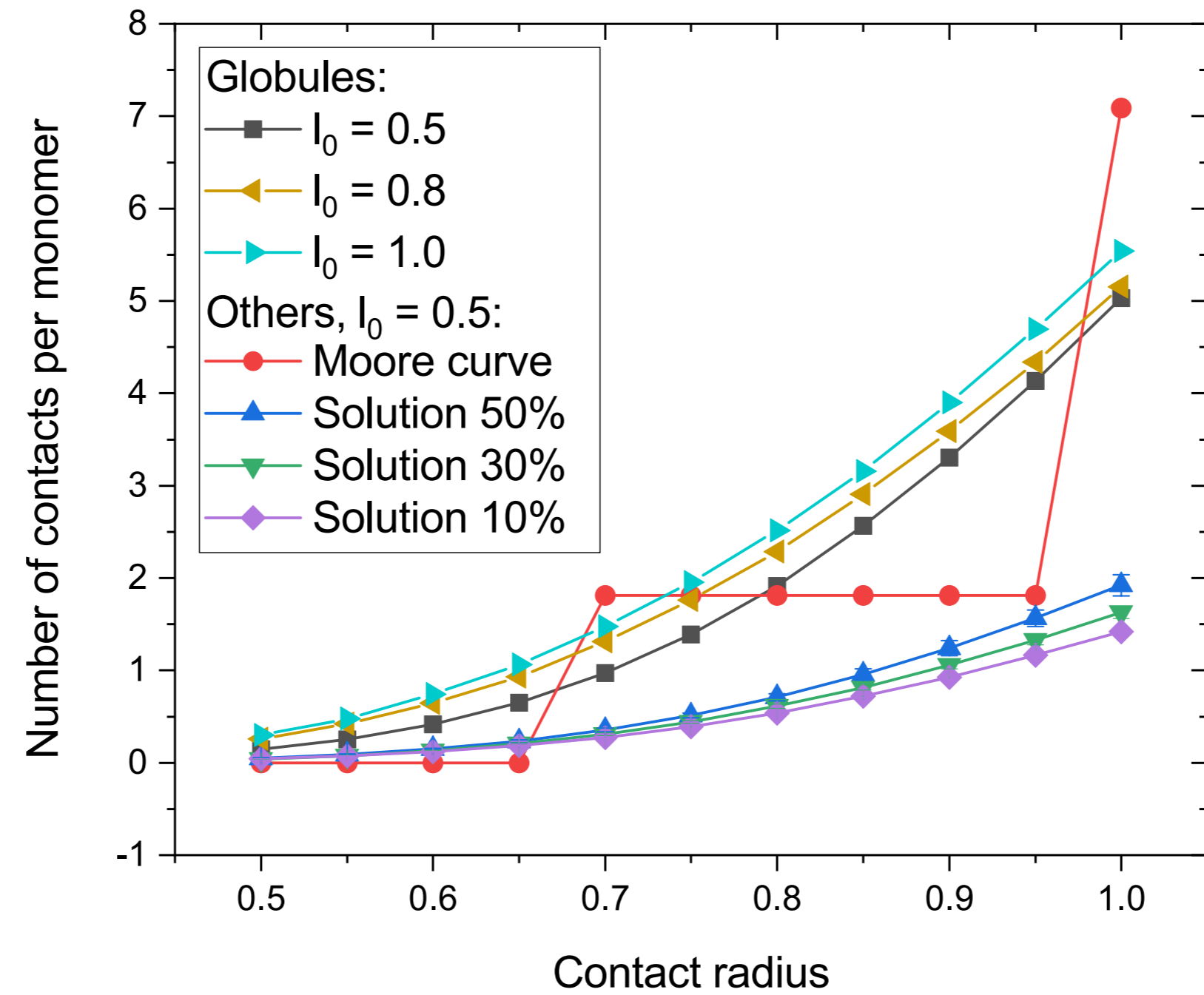

Supplement: S6 Fig — Dependence of specific number of contacts per polymer bead on cutting radius for the systems with various polymer concentration and for globule with various initial bond length. (PDF) [file pcbi.1009546.s006.pdf]

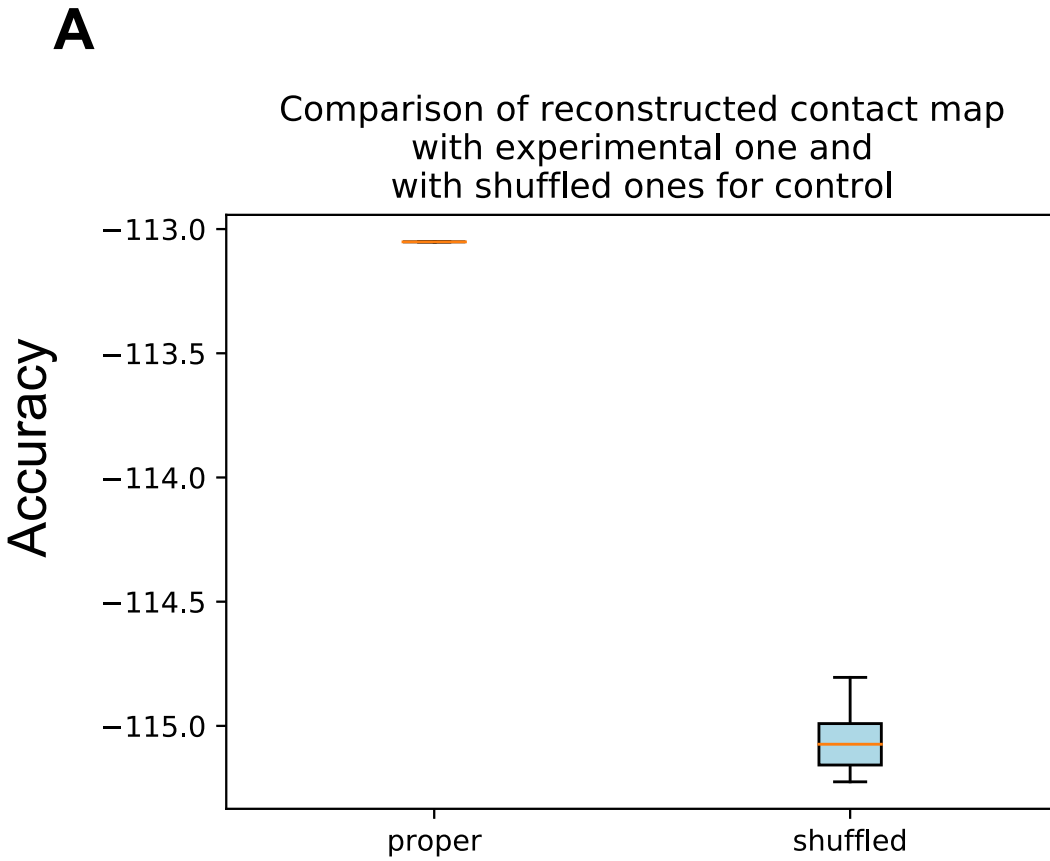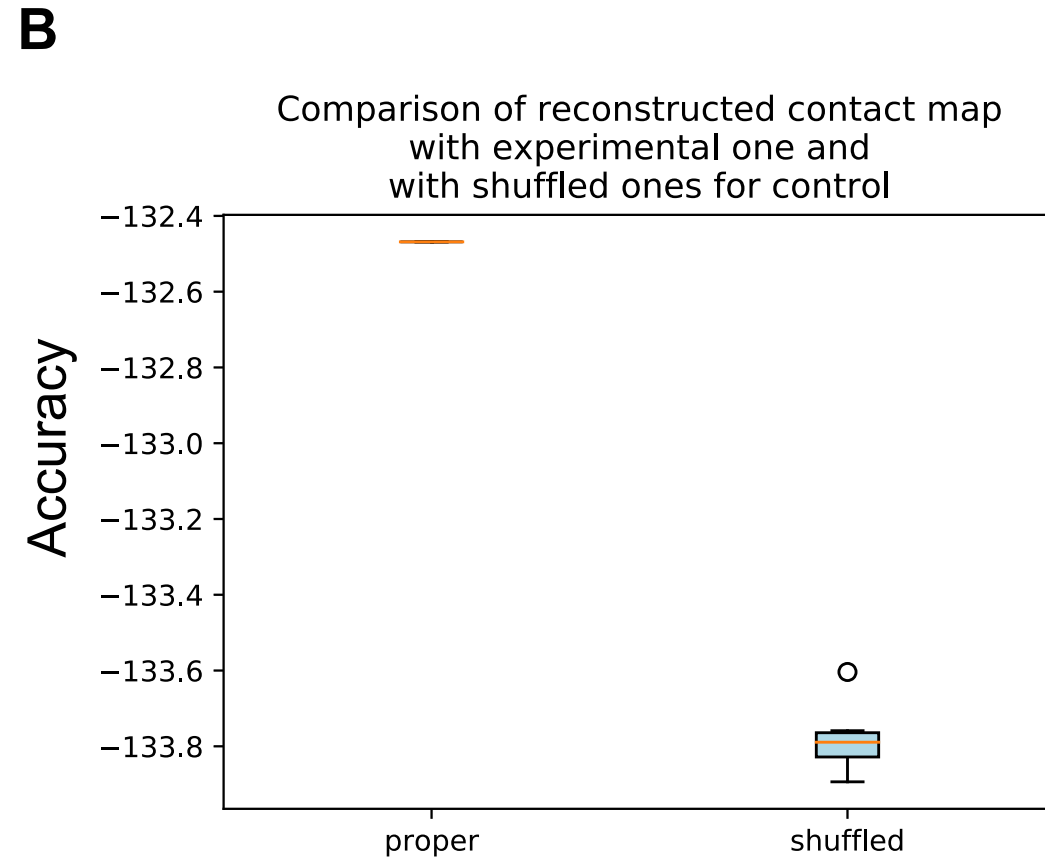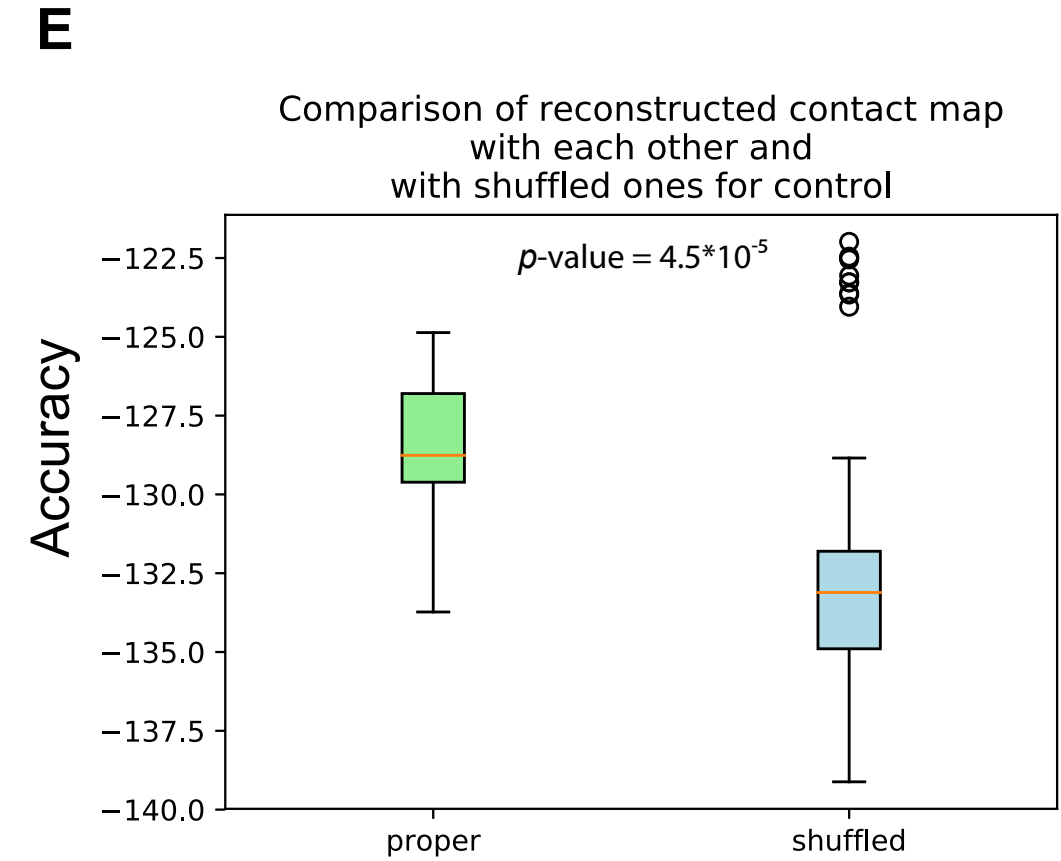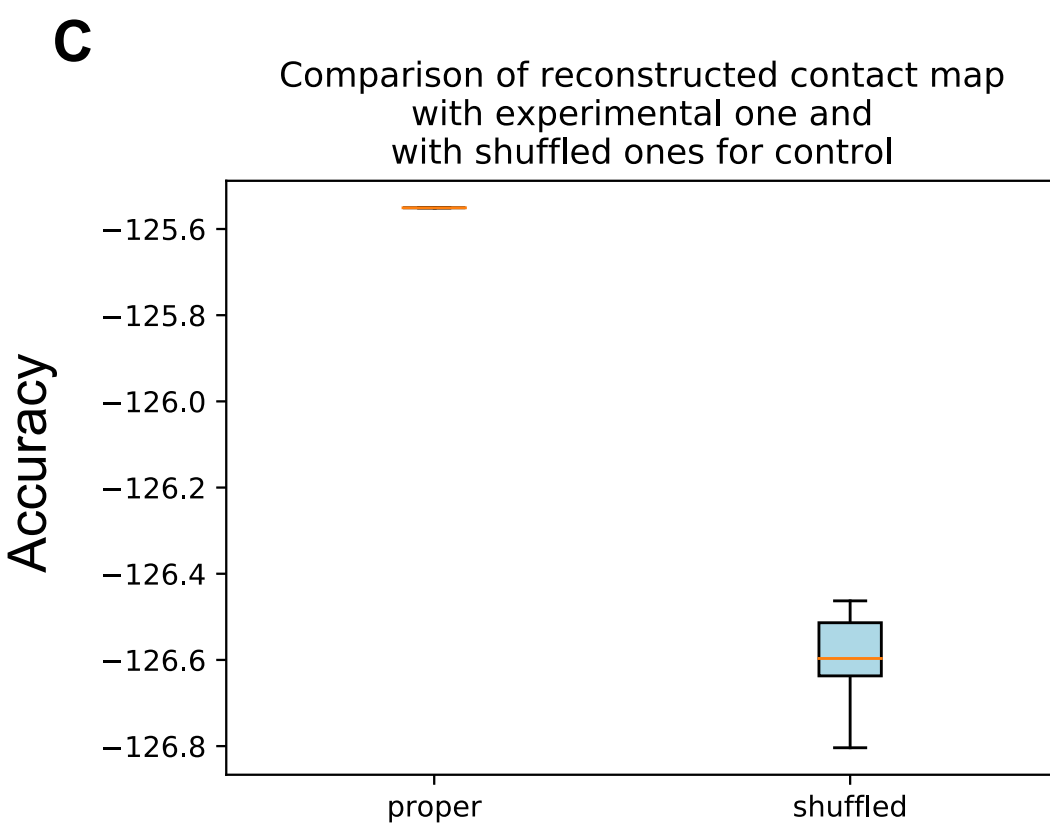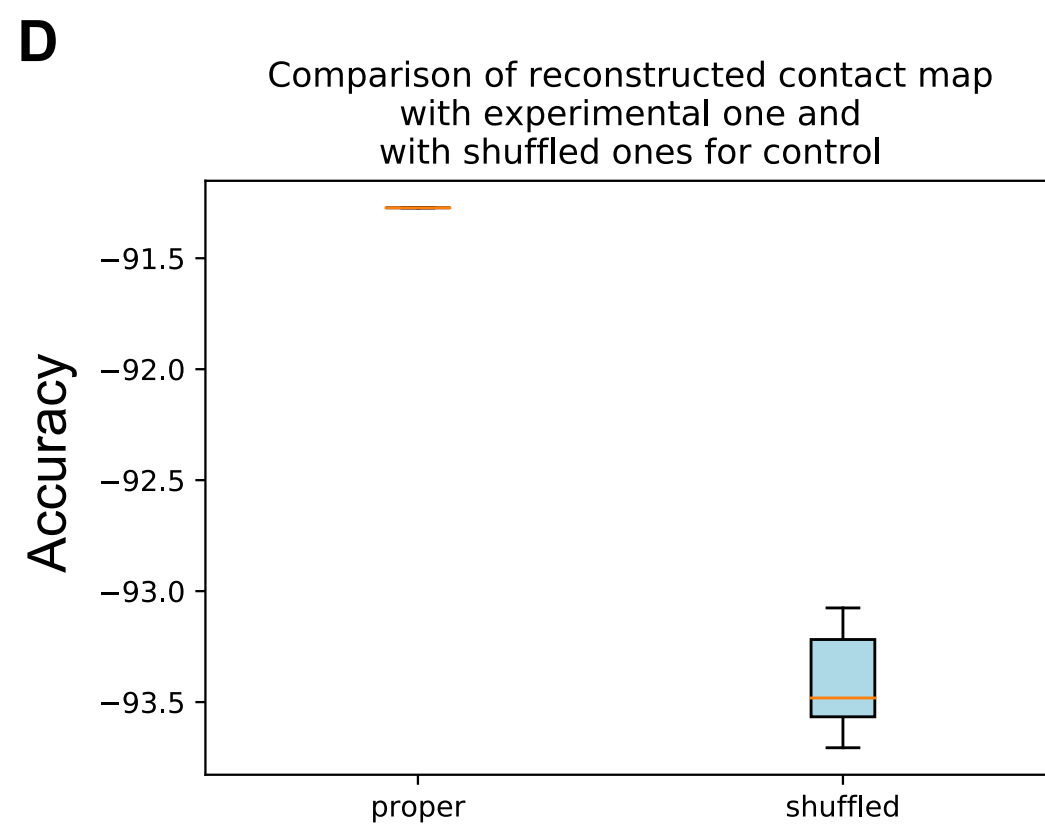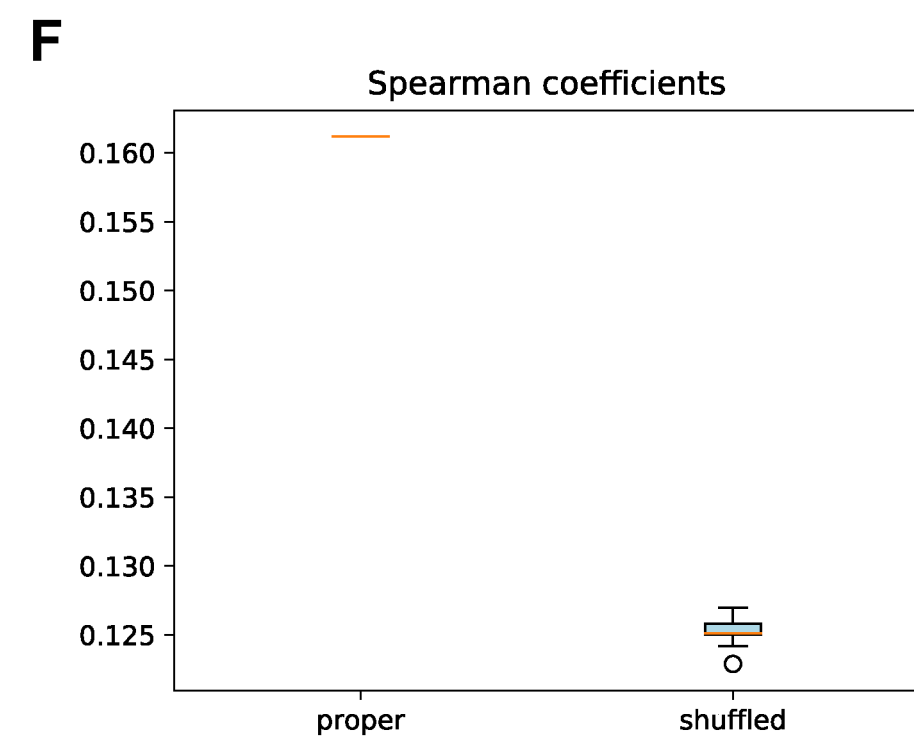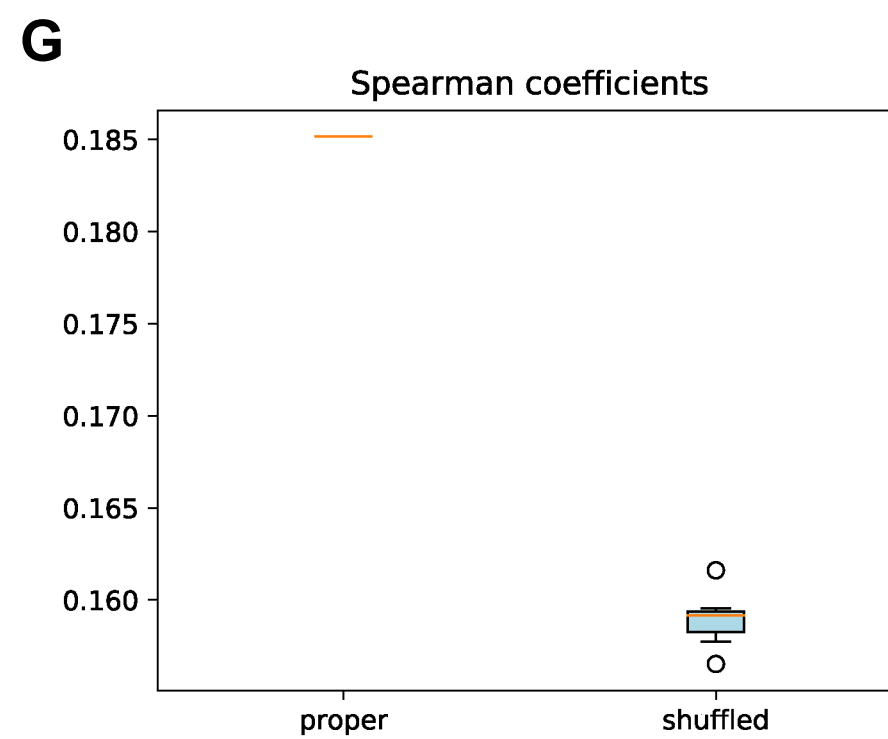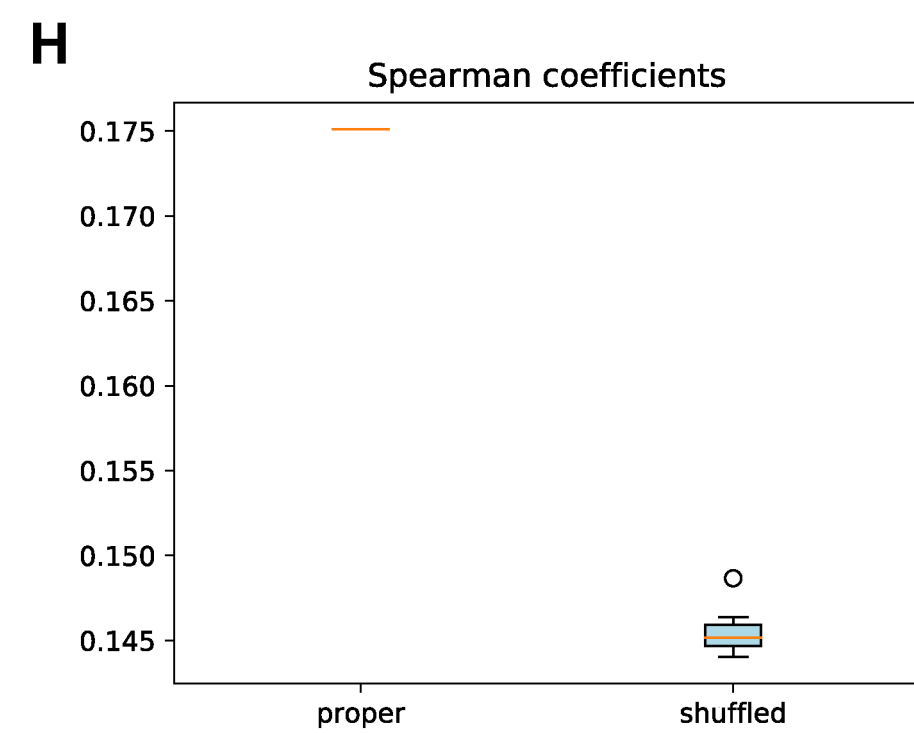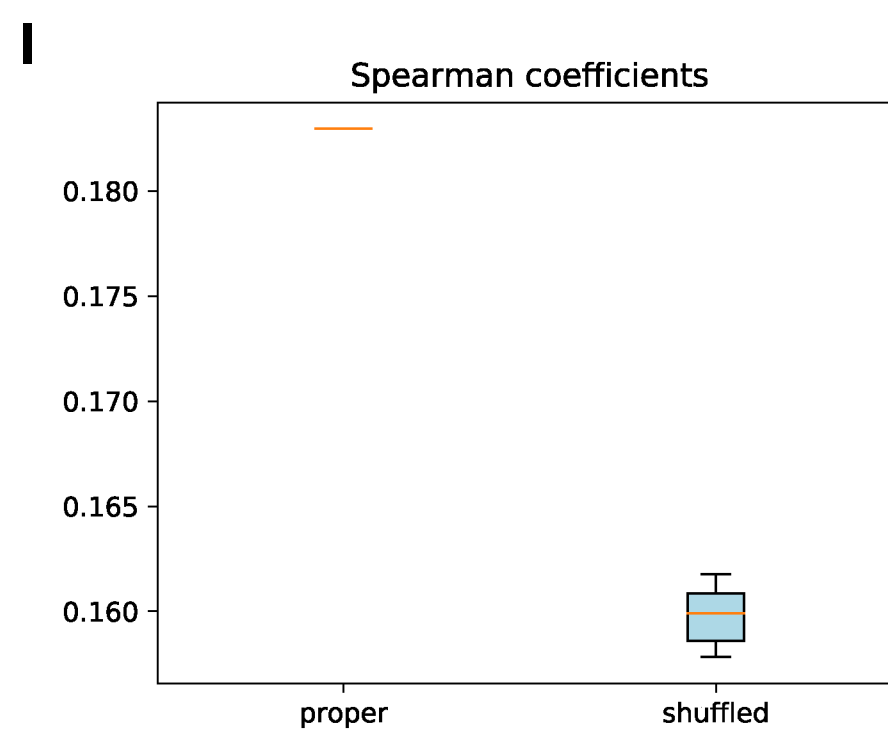

Supplement: S7 Fig — Accuracies were calculated according to the definition from The Modified Jaccard Index. A-D. “Proper” means the similarity of the contact map of reconstructed chromosome 4 from the cells 1 − 4 and merged experimental contact map of all datasets. “Shuffled” means the similarity of the shuffled contact map of reconstructed chromosome 4 from the cells 1 − 4, keeping the initial amount of contacts on the sub-diagonal, and merged experimental contact map. E. “Proper” corresponds to the similarity of contact maps of reconstructed conformations to each other. “Shuffled” corresponds to the similarity of contact maps of the reconstructed conformations to shuffled ones. Shuffled were prepared in the same way as in a-d. Group of the outliers correspond to the cell 4. Its contact maps is more similar to the shuffled one than to other cells. F-I. The same as (A-D) but instead of IMJ we used the Spearman correlation coefficient. (PDF) [file pcbi.1009546.s007.pdf]

A

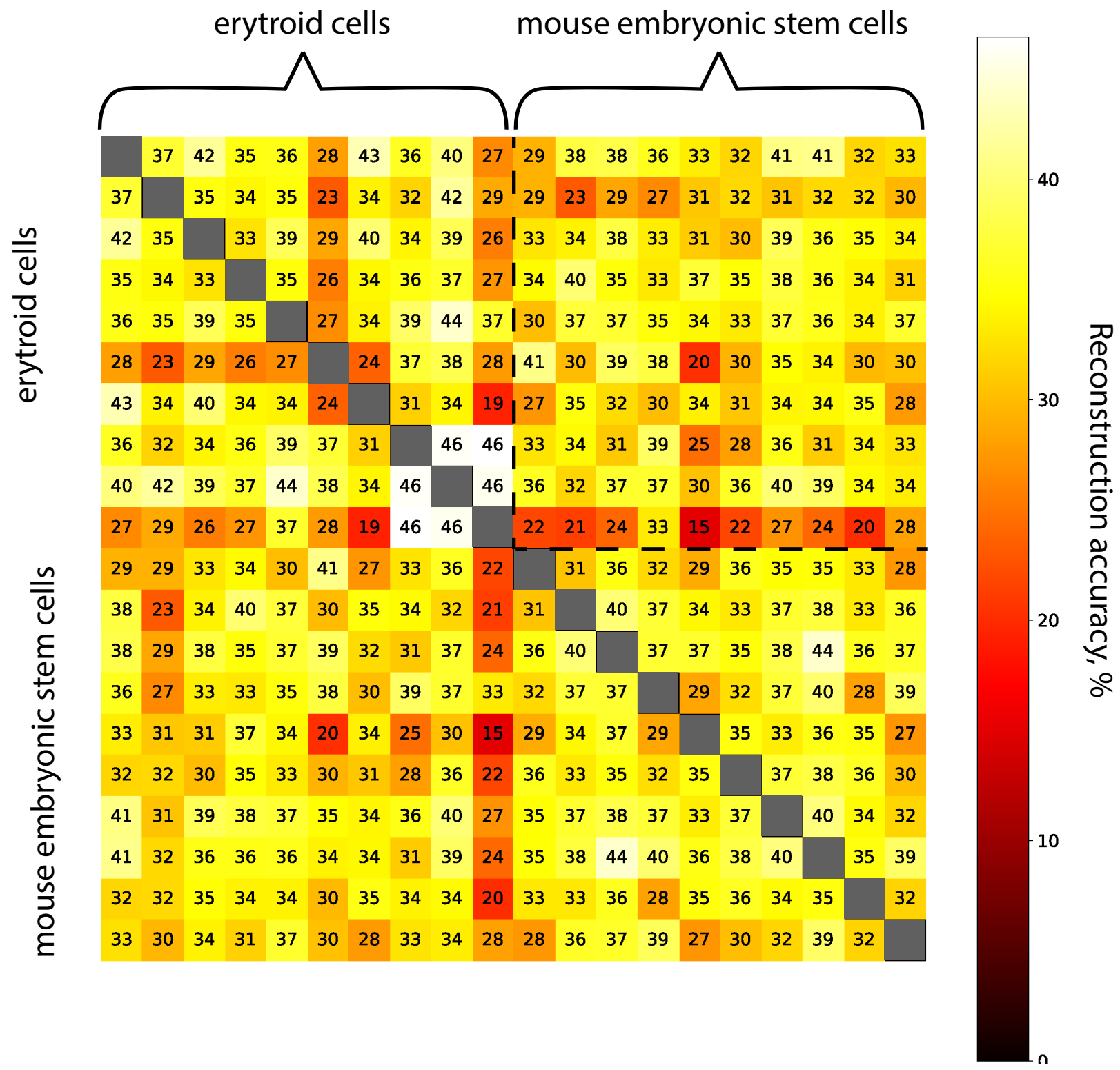

B

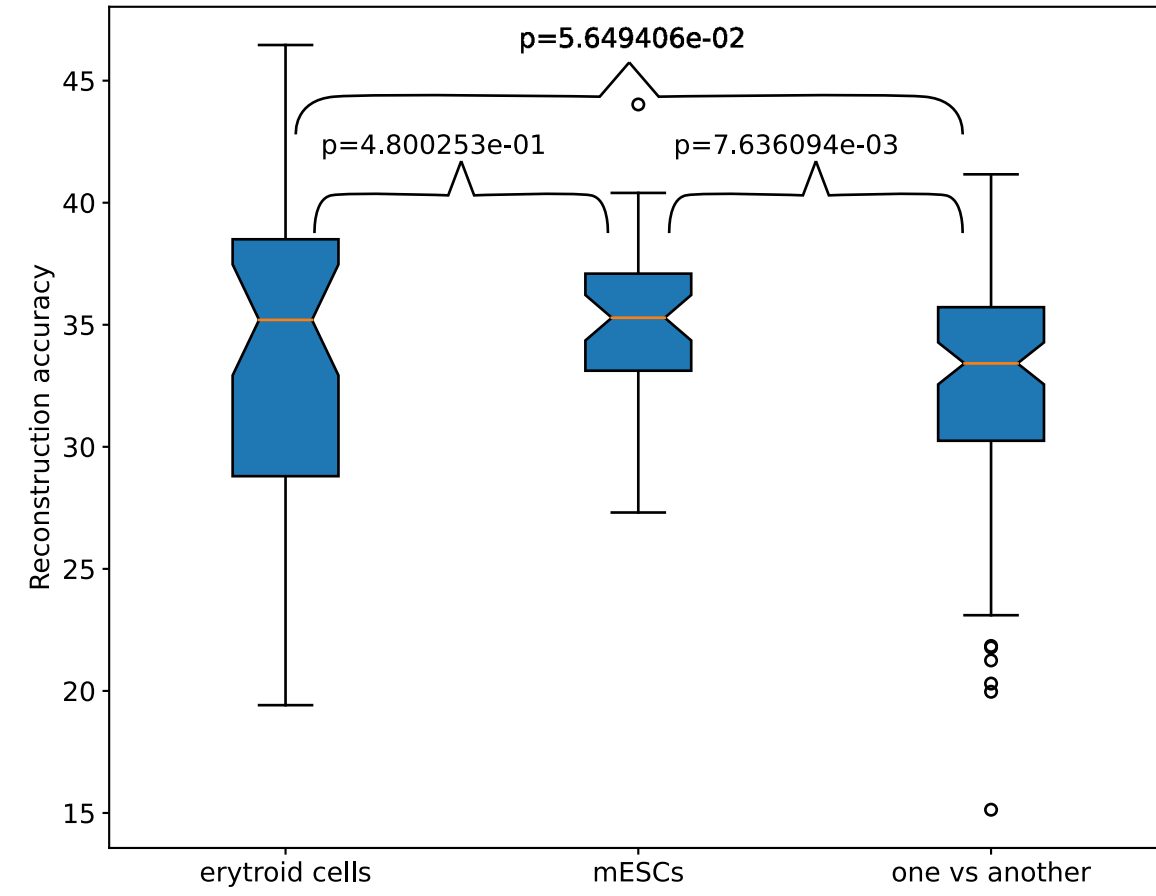

C

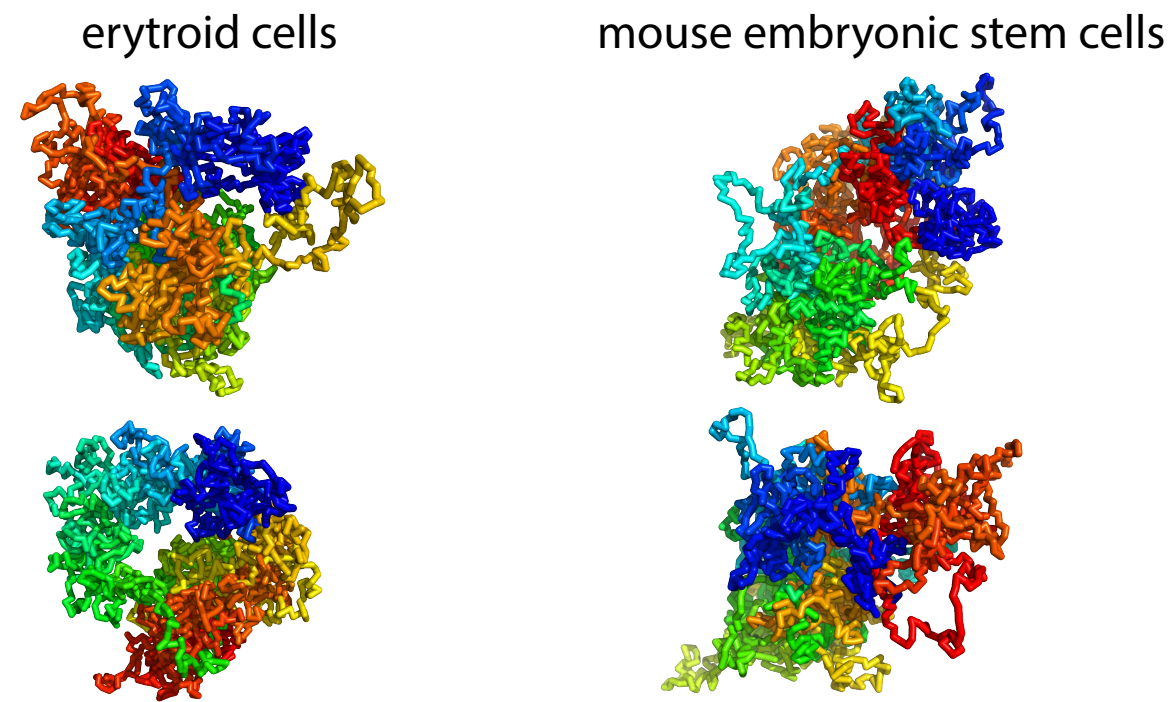

D

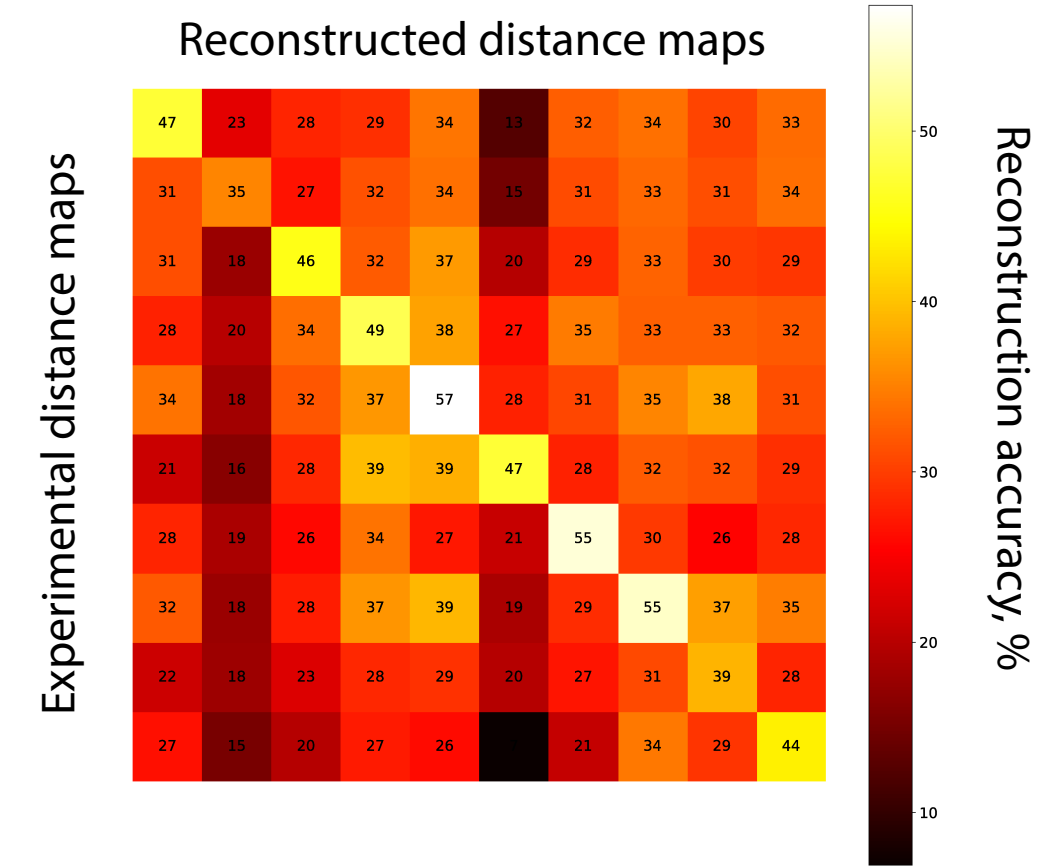

E

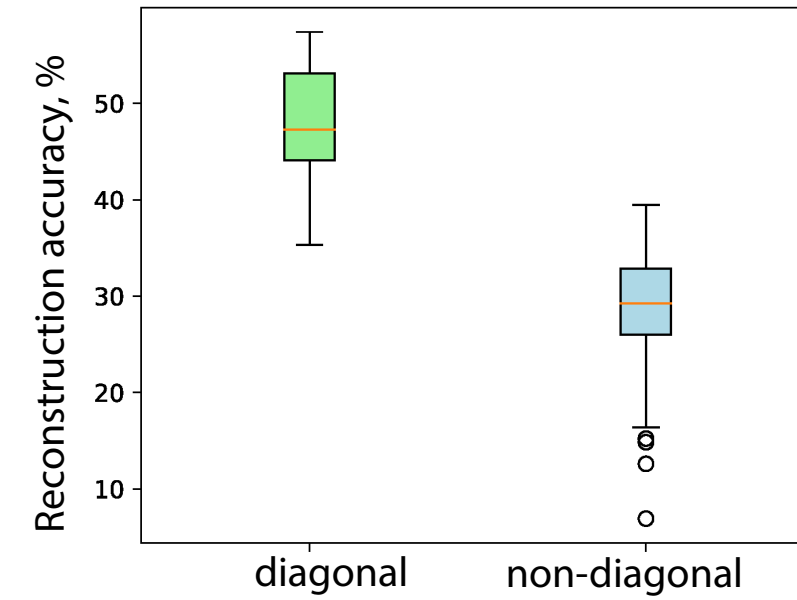

F

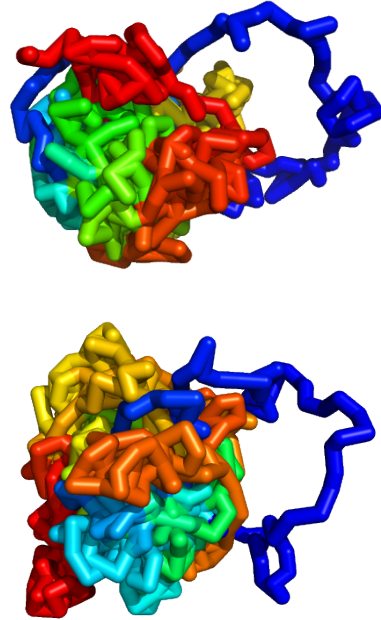

Supplement: S8 Fig — A. Pairwise comparison of distance maps corresponding to experimental capture-C data [30, 31] and reconstructed 3D model. In a cell unit, a number indicates reconstruction accuracy, %. B. Summary of matrix from (A). Boxplots of reconstruction accuracies. p-values correspond to the two sample KS test. C. Examples of reconstructed 3D conformations of ECs and mESCs. D. Pairwise comparison of distance maps corresponding to experimental oligopaint data [33] corresponding to chromosome 21. E. Boxplots of reconstruction accuracies for (D), similar to (B). F. Examples of two reconstructed 3D structures from (D). (PDF) [file pcbi.1009546.s008.pdf]
