# Supplementary material for: Perspectives for the reconstruction of 3D chromatin conformation using single cell Hi-C data
Source: PLoS Comput Biol. 2021 Nov 18;17(11):e1009546. doi: 10.1371/journal.pcbi.1009546 (PMC8601426; doi:10.1371/journal.pcbi.1009546)
Supplement: S1 Table — Kolmogorov-Smirnov tests for DPD and CMD distributions from Fig 2D and 2E. (PDF) [file pcbi.1009546.s009.pdf]

S1 Table. Kolmogorov-Smirnov test for DPD and CMD distributions in Fig. 2 d,e.

	1-2	1-3	2-3
DPD	0.88	0.56	0.94
CMD	0.0	0.0	0.0004
