# Supplementary material for: Perspectives for the reconstruction of 3D chromatin conformation using single cell Hi-C data
Source: PLoS Comput Biol. 2021 Nov 18;17(11):e1009546. doi: 10.1371/journal.pcbi.1009546 (PMC8601426; doi:10.1371/journal.pcbi.1009546)
Supplement: S2 Table — (PDF) [file pcbi.1009546.s010.pdf]

S2 Table. List of single nucleus Hi-C datasets.

Number of dataset	Name in the paper or GEO
1	oocyte intermediate [1]
2	oocyte intermediate Hoechst [1]
3	oocyte non-surrounding nucleolus [1]
4	oocyte non-surrounding nucleolus Hoechst [1]
5	oocyte surrounding nucleolus [1]
6	oocyte surrounding nucleolus Hoechst [1]
7	G2 [2]
8	Scc1 control + Scc1 flox [2]
9	Scc1 KO [2]
10	Wapl control + Wapl flox [2]
11	Wapl KO [2]

References

[1] IM Flyamer, et al., Single-nucleus hi-c reveals unique chromatin reorganization at oocyte-to-zygote transition. *Nature* **544**, 110 (2017).

[2] J Gassler, et al., A mechanism of cohesin-dependent loop extrusion organizes zygotic genome architecture. *The EMBO journal* **36**, 3600–3618 (2017).
