# Supplementary material for: Perspectives for the reconstruction of 3D chromatin conformation using single cell Hi-C data
Source: PLoS Comput Biol. 2021 Nov 18;17(11):e1009546. doi: 10.1371/journal.pcbi.1009546 (PMC8601426; doi:10.1371/journal.pcbi.1009546)
Supplement: S1 Appendix — Description of DPD, CMD, Stevens method, insights to polymer solutions, information on statistical tests, description of single-nucleus Hi-C data processing, and ORBITA protocol. (PDF) [file pcbi.1009546.s011.pdf]

Supporting information to "Perspectives for the reconstruction of 3D chromatin conformation using single cell Hi-C data"

P.I. Kos, A.A. Galitsyna,
S.V. Ulyanov, M.S. Gelfand,
S.V. Razin, A.V. Chertovich

October 23, 2021

S1 Appendix

Classic molecular dynamics

This method allows to study physical properties of liquids and polymers by solving numerically Newton's equations of motion at each simulation time step [10]. The Lennard-Jones potential Eq. 1 and the potential energy of harmonic oscillator Eq. 1 describe the non-bonded and bonded interactions, respectively.

The resolution of single cell Hi-C is usually worse than 1 Kb. The correlation of directions between the beads is not expected (persistent length assumed to be less than 1 Kb) [1]. Thus, we did not introduce potentials on angles and dihedral angles. On the other hand, that the polymer chain is not a freely-jointed one because beads have excluded volume.

$$U_{LJ} = \begin{cases} 4\epsilon\Big(\Big(\frac{\sigma^{CMD}}{r_{ij}}\Big)^{12} - \Big(\frac{\sigma^{CMD}}{r_{ij}}\Big)^{6}\Big), & r_{ij} < r_c^{CMD} \\ 0, \quad r_{ij} \geq r_c^{CMD} \end{cases} \tag{1}$$

$$U_{bond} = k^{CMD}(r_{ij} - l_0^{CMD})^2 \tag{2}$$

In Eq. 1 ϵ is the depth of potential well, σ^{CMD} is the particle size and r_c^{CMD} is the cutting radius; we fixed two latter parameters. Depending on the well's depth ϵ^{CMD}, the solvent is good, neutral, or poor, *e.i.* a polymer chain forms a swollen coil, a random walk, or a globule, respectively (Appendix in SI). In Eq. 2, k^{CMD} is the spring stiffness constant, l_0^{CMD} is the free length of a spring, and r_{ij} is the 3D distance between beads i and j.

To perform classic molecular dynamics simulations, we used a standard open source package LAMMPS [2]. The input scripts to reproduce the simulations are available at github [3].

Dissipative particle dynamics

DPD is a coarse-grain molecular dynamics simulation method [9]. The initial method DPD was proposed by P. J. Hoogerbrugge and J. M. V. A. Koelman [6, 7]. Later it was modified to consider continuous system with different weight functions for dissipative and random forces [8, 9]. Similar to CMD, we solve Newton's equations of motions at each simulation step for each particle. The force consists of four terms: the conservative force of soft repulsion Eq. 3, the dissipative force of viscous friction Eq. 4, the paired random force (heat generator) Eq. 5, and the elastic force

between connected beads (bonds) Eq. 6.

$$\vec{F_{ij}^{C}} = \begin{cases} a_{ij}(r_c^{DPD} - r_{ij})\frac{\vec{r_{ij}}}{|\vec{r_{ij}}|}, & r_{ij} < r_c^{DPD} \\ 0, & r_{ij} \geq r_c^{DPD} \end{cases} \tag{3}$$

$$\vec{F_{ij}^{D}} = -\gamma w^D(r_{ij})\Big(\frac{\vec{r_{ij}}}{|\vec{r_{ij}}|}\vec{v_{ij}}\Big)\frac{\vec{r_{ij}}}{|\vec{r_{ij}}|}, \quad r_{ij} < r_c^{DPD} \tag{4}$$

$$\vec{F_{ij}^{R}} = \sigma w^R(r_{ij})\theta_{ij}\frac{\vec{r_{ij}}}{|\vec{r_{ij}}|}, \quad r_{ij} < r_c \tag{5}$$

$$\vec{F_{ij}^{B}} = k^{DPD}(r_{ij} - l_0^{DPD}) \tag{6}$$

In Eq. 3, a_{ij} is the maximum repulsion between beads i and j, r_c^{DPD} is the cutting radius, r_{ij} is the distance between beads i and j. In Eq. 4, γ is the friction coefficient, w^D is the weight function, and v_{ij} is the relative velocity between beads i and j. In Eq. 5, σ is the noise level, w^R is the weight function, and θ_{ij} is the Gaussian white-noise term. In Eq. 6, k^{DPD} is the spring stiffness constant, r_{ij} is the bond length between the beads i and j, and l_0^{DPD} is the free length of a spring (bond).

The elastic force potential is similar to that in CMD, Eq. 2. Interaction with the solvent depends on the difference of the repulsion coefficients of conservative force $\Delta a = a_{ij} - a_{ii}$, where a_{ii} is the repulsion coefficient between beads of the same type (that is, polymer and polymer or solvent and solvent), and a_{ij} is the repulsion coefficient between beads of different types (that is, polymer and solvent). Parameter Δa may be converted into Flory-Huggins parameter as $\chi = 0.306\Delta a$ in the case of polymer chain [9]. Similar to CMD, the conditions for a good, neutral, or poor solvent can also be specified in DPD.

$$\vec{F_{ij}^{D}} = -\gamma w^D(r_{ij})\Big(\frac{\vec{r_{ij}}}{|\vec{r_{ij}}|}\vec{v_{ij}}\Big)\frac{\vec{r_{ij}}}{|\vec{r_{ij}}|}, \quad r_{ij} < r_c^{DPD} \tag{7}$$

$$\vec{F_{ij}^{R}} = \sigma w^R(r_{ij})\theta_{ij}\frac{\vec{r_{ij}}}{|\vec{r_{ij}}|}, \quad r_{ij} < r_c \tag{8}$$

The forces from Eq. 7 and Eq. 8 constitute the thermostat of the DPD method. The heat generator is compensated by the viscous friction. w^D and w^R are weight functions, γ is a friction coefficient, σ is a noise level and θ_{ij} is a Gaussian white-noise term. The w^D, w^R, γ and σ compose fluctuation-dissipation theorem for DPD, see Eq. 9, 10, where k_B is the Boltzmann constant and T is the temperature. The theorem has the same view as for the Brownian dynamics.

$$w^R(r) = [w^D(r)]^{1/2} \tag{9}$$

$$\sigma = (2k_BT\gamma)^{1/2} \tag{10}$$

The Gaussian white-noise θ_{ij} satisfy the conditions in Eq. 11-13.

$$\theta_{ij} = \theta_{ji} \tag{11}$$

$$\langle\theta_{ij}(t)\rangle = 0 \tag{12}$$

$$\langle\theta_{ij}(t)\theta_{kl}(t')\rangle = (\delta_{ik}\delta_{jl} + \delta_{il}\delta_{jk})\delta(t - t') \tag{13}$$

The random force is paired (Eq. 11) and applied along the straight line connecting the particles so the total momentum is conserved at every single intergration step.

$$d(\sum_i \vec{p_i})/dt = 0 \tag{14}$$

The integration scheme is implemented as a modified version of the velocity-Verlet algorithm [10].

To perform DPD calculations, we used our own implementation of dissipative particle dynamics, MPI parallel version. The code and examples are available on github [3].

The Stevens method

The idea of this annealing protocol is to minimize the energy hierarchically [4]. One should specify the resolution steps, the default set is 8 Mb, 2 Mb, 1 Mb, and 500 Kb, and the number of alternative models, the default value is 10.

Energy of the system consists of non-adjacent and adjacent terms. The former represents soft repulsion between the non-adjacent beads, Eq. 15. The latter represents bond stretching using non-trivial force, Eq. 17.

$$F_{rep} = \begin{cases} k_1(d - d_{lim})^2, & d < d_{lim} \\ 0, & d \geq d_{lim} \end{cases} \tag{15}$$

$$k_1 = 0.5 + \frac{1}{\pi tan(\alpha)} tan^{-1}(\alpha(2x-1)) \tag{16}$$

$$F_{dist} = \begin{cases} k_2(d - d_{lower})^2, & d < d_{lower} \\ 0, & d_{lower} \leq d \leq d_{upper} \\ -k_2(d - d_{upper})^2, & d_{upper} < d < d_{upper} + 0.5 \\ -k_2(d_{upper} - d + 0.25), & d \geq d_{upper} + 0.5 \end{cases} \tag{17}$$

The temperature is defined via kinetic energy. One should define the starting temperature, the final temperature, the temperature step, and the number of simulation steps. The integration time step equals 0.001, which is similar to the CMD approach. The choice of the rest of parameters ($k_1, k_2, d_{lim}, d_{upper}, d_{lower}$, etc.) is described in Supplementary materials in the original paper [4].

To convert data to a format for the Stevens method there is a script rst2ncc.py in repository [3]. Output of the Stevens method was converted to .xyz files. These files were compared with the standard restart files from DPDchrom using script compare_ncc_w_rst.py from repository [3].

Polymer solution

One of the common ways to describe the mixture of homopolymer chain and solvent is the Flory-Huggins solution theory.

$$\Delta G_m = RT(n_1 \cdot log\phi_1 + n_2 \cdot log\phi_2 + n_1 \cdot \phi_2 \cdot \chi_{12}) \tag{18}$$

where G is the Gibbs energy, R is the gas constant, T is the temperature, n_1 and n_1 are the moles of components 1 and 2, ϕ_1 and ϕ_2 are the volume fractions of components 1 and 2 and χ_{12} is the Flory-Huggins parameter.

The Flory-Huggins parameter $\chi = 1/2$ corresponds to the θ-solvent, it means that a polymer chain in a dilute solution behaves like an ideal (Gaussian) chain, spatial distance between any two beads separated by s beads is proportional to the square root of s ($R(s) \sim s^{1/2}$) [5]. If the Flory-Huggins parameter is greater than 1/2, then the solvent is poor and chain tend to form a globule having $R(s) \sim s^{1/2}$, if $s \leq s^{2/3}$ and $R(s) \sim const$, if $s > s^{2/3}$. In our simulations, we used $\chi = 9$ to simulate poor solvent. The Flory-Huggins parameter $\chi = 0$ corresponds to the good solvent, resulting in sparse polymer structure with $R(s) \sim s^{3/5}$.

Statistical tests

We performed Kolmogorov-Smirnov tests to show significance of differences between bond length distributions, see Fig 2D and 2E. Let's denote 1 is the bond length distribution of the gold-standard model, 2 is the backbone bond length distribution of the reconstructed model, and 3 is the additional bond length distribution of the reconstructed model, S1 Table.

Kolmogorov-Smirnov test [11] shows that for DPDchrom p-value for the distributions 1 and 2 is 0.88, for distributions 1 and 3 is 0.56, and for distributions 2 and 3 is 0.94. Respectively for CMD, p-value for the distributions 1 and 2 is 0.0, for distributions 1 and 3 is 0.0, and for distributions 1 and 3 is 0.0004.

Single-nucleus Hi-C data processing

For tesing on experimental data, we downloaded 11 single-nucleus Hi-C datasets [12, 13] (S2 Table) and performed *de novo* analysis with the One-Read Based Interactions Annotation (ORBITA) approach [14].

Briefly, in this approach we keep the single-cell Hi-C contacts that are supported by restriction junction between chimeric parts and originate from fragments with at most five unique contacts. This decreases the noise in the data such as random hops of polymerase.

ORBITA protocol

In order to filter out artifact snHi-C chimeras arising from hops of polymerase, genomic rearrangements and random DNA breaking, we performed snHi-C re-analysis with ORBITA (One Read-Based Interaction Annotation) approach [14]. In this approach we considered only the reads traversing restriction sites as true contacts.

Mapping of reads was done with bwa mem [15] at mm9 mouse genome assembly. Reads were processed with adapted version of pairtools [16] with ORBITA parsing option. For each chimeric part we annotated the corresponding restriction fragment end. We then removed the amplificated pairs by retaining only the unique pairs of restriction fragments interactions. Number of contacts per each restriction fragment end was assessed in each experiment, only the fragments with less than 4 unique contacts were retained. Maps were constructed with cooler at 10 Kb resolution [17].

We performed *de novo* mapping of snHi-C murine datasets from [12, 13]. For assessment of number of contacts per monomer unit, we combined all the datasets from the same cell type and treatment. We obtained 11 groups in total.

References

[1] Redolfi, J., Zhan, Y., Valdes-Quezada, C., Kryzhanovska, M., Guerreiro, I., Iesmantavicius, V., Pollex, T., Grand, R. S., Mulugeta, E., Kind, J., et al. (2019) DamC reveals principles of chromatin folding in vivo without crosslinking and ligation. *Nature structural & molecular biology,* **26**(6), 471–480.

[2] Plimpton, S. (1995) Fast parallel algorithms for short-range molecular dynamics. *Journal of computational physics,* **117**(1), 1–19.

[3] Kos, P. Dissipative Particle Dynamics for chromatin modeling. `https://github.com/polly-code/DPDchrom` (2021).

[4] Stevens, T. J., Lando, D., Basu, S., Atkinson, L. P., Cao, Y., Lee, S. F., Leeb, M., Wohlfahrt, K. J., Boucher, W., O'Shaughnessy-Kirwan, A., et al. (2017) 3D structures of individual mammalian genomes studied by single-cell Hi-C. *Nature,* **544**(7648), 59.

[5] PJ Flory, M Volkenstein, Statistical mechanics of chain molecules. *Biopolymers: Original Research on Biomolecules* **8**, 699–700 (1969).

[6] P Hoogerbrugge, J Koelman, Simulating microscopic hydrodynamic phenomena with dissipative particle dynamics. *EPL (Europhysics Letters)* **19**, 155 (1992).

[7] J Koelman, P Hoogerbrugge, Dynamic simulations of hard-sphere suspensions under steady shear. *EPL (Europhysics Letters)* **21**, 363 (1993).

[8] P Espanol, P Warren, Statistical mechanics of dissipative particle dynamics. *EPL (Europhysics Letters)* **30**, 191 (1995).

[9] RD Groot, PB Warren, Dissipative particle dynamics: Bridging the gap between atomistic and mesoscopic simulation. *The Journal of chemical physics* **107**, 4423–4435 (1997).

[10] MP Allen, DJ Tildesley, *Computer simulation of liquids.* (Oxford university press), (2017).

[11] FJ Massey Jr, The kolmogorov-smirnov test for goodness of fit. *Journal of the American statistical Association* **46**, 68–78 (1951).

[12] IM Flyamer, et al., Single-nucleus hi-c reveals unique chromatin reorganization at oocyte-to-zygote transition. *Nature* **544**, 110 (2017).

[13] J Gassler, et al., A mechanism of cohesin-dependent loop extrusion organizes zygotic genome architecture. *The EMBO journal* **36**, 3600–3618 (2017).

[14] A Galytsyna, Orbita (`https://github.com/agalitsyna/pairtools`) (2020).

[15] L H., D R., Fast and accurate short read alignment with burrows-wheeler transform. *Bioinformatics* **25**, 1754–60 (2009).

[16] Adapted version of pairtools (`https://github.com/mirnylab/pairtools/tree/master/pairtools`) (2020).

[17] LAM Nezar Abdennur, Cooler: scalable storage for hi-c data and other genomically labeled arrays. *Bioinformatics* **btz540** (2019).
